# Heme Induces Endoplasmic Reticulum Stress (HIER Stress) in Human Aortic Smooth Muscle Cells

**DOI:** 10.3389/fphys.2018.01595

**Published:** 2018-11-20

**Authors:** Tamás Gáll, Dávid Pethő, Annamária Nagy, Zoltán Hendrik, Gábor Méhes, László Potor, Magnus Gram, Bo Åkerström, Ann Smith, Péter Nagy, György Balla, József Balla

**Affiliations:** ^1^HAS-UD Vascular Biology and Myocardial Pathophysiology Research Group, Hungarian Academy of Sciences, Debrecen, Hungary; ^2^Department of Pediatrics, Faculty of Medicine, University of Debrecen, Debrecen, Hungary; ^3^Department of Internal Medicine, Faculty of Medicine, University of Debrecen, Debrecen, Hungary; ^4^Department of Pathology, Faculty of Medicine, University of Debrecen, Debrecen, Hungary; ^5^Department of Clinical Sciences Lund, Infection Medicine, Lund University, Lund, Sweden; ^6^Department of Molecular Biology and Biochemistry, School of Biological Sciences, University of Missouri-Kansas City, Kansas City, MO, United States; ^7^Department of Vascular Surgery, Faculty of Medicine, University of Debrecen, Debrecen, Hungary

**Keywords:** heme, vascular smooth muscle cell, heme induced endoplasmic reticulum stress, atherosclerosis, hemopexin, alpha-1-microglobulin

## Abstract

Accumulation of damaged or misfolded proteins resulted from oxidative protein modification induces endoplasmic reticulum (ER) stress by activating the pathways of unfolded protein response. In pathologic hemolytic conditions, extracellular free hemoglobin is submitted to rapid oxidation causing heme release. Resident cells of atherosclerotic lesions, after intraplaque hemorrhage, are exposed to heme leading to oxidative injury. Therefore, we raised the question whether heme can also provoke ER stress. Smooth muscle cells are one of the key players of atherogenesis; thus, human aortic smooth muscle cells (HAoSMCs) were selected as a model cell to reveal the possible link between heme and ER stress. Using immunoblotting, quantitative polymerase chain reaction and immunocytochemistry, we quantitated the markers of ER stress. These were: phosphorylated eIF2α, Activating transcription factor-4 (ATF4), DNA-damage-inducible transcript 3 (also known as C/EBP homology protein, termed CHOP), X-box binding protein-1 (XBP1), Activating transcription factor-6 (ATF6), GRP78 (glucose-regulated protein, 78kDa) and heme responsive genes heme oxygenase-1 and ferritin. In addition, immunohistochemistry was performed on human carotid artery specimens from patients who had undergone carotid endarterectomy. We demonstrate that heme increases the phosphorylation of eiF2α in HAoSMCs and the expression of ATF4. Heme also enhances the splicing of XBP1 and the proteolytic cleavage of ATF6. Consequently, there is up-regulation of target genes increasing both mRNA and protein levels of CHOP and GRP78. However, TGFβ and collagen type I decreased. When the heme binding proteins, alpha-1-microglobulin (A1M) and hemopexin (Hpx) are present in cell media, the ER stress provoked by heme is inhibited. ER stress pathways are also retarded by the antioxidant N-acetyl cysteine (NAC) indicating that reactive oxygen species are involved in heme-induced ER stress. Consistent with these findings, elevated expression of the ER stress marker GRP78 and CHOP were observed in smooth muscle cells of complicated lesions with hemorrhage compared to either atheromas or healthy arteries. In conclusion, heme triggers ER stress in a time- and dose-dependent manner in HAoSMCs. A1M and Hpx as well as NAC effectively hamper heme-induced ER stress, supporting their use as a potential therapeutic approach to reverse such a deleterious effects of heme toxicity.

## Introduction

Vascular diseases including atherosclerosis remain one of the major causes of death worldwide (Roth et al., 2017). Atherosclerosis is associated with various pathological manifestations like ischemic heart disease, ischemic stroke, and peripheral arterial disease remaining the leading public health issue (Herrington et al., 2016). A more complete understanding of the pathophysiology would lead to new interventions that are urgently needed especially to treat the advanced, irreversible stage of atherosclerosis. Type IV atherosclerotic lesions, which are characterized by pronounced extracellular lipid accumulation, easily progress into more complicated lesions by the rupture of the fibrous cap and subsequent hematoma formation or intraplaque hemorrhage (Barger et al., 1984; Stary et al., 1995; Kolodgie et al., 2003; Moreno et al., 2006, 2012). The highly oxidative environment of these complicated lesions contain lipid peroxidation products such as lipid hydroperoxides, oxysterols, aldehydes and carbonyls, which are extremely toxic to cells of the vascular wall. Our group has been interested in this area, especially the role of heme in the pathogenesis of plaque progression (Balla et al., 2007).

In cell free models, heme was able to cause lipid peroxidation characterized by extensive cross-linking with subsequent amino acid oxidation (Gutteridge and Smith, 1988; Vincent, 1989). Moreover, heme catalyzes the degradation of proteins to small peptide fragments (Aft and Mueller, 1984). Our group was the first in the literature providing evidence that free heme, having amphipathic nature, can enter to lipid domains of living cells sensitizing them toward any types of reactive oxygen species (ROS) derived from activated neutrophils or inorganic-, organic hydroperoxides leading to cell death (Balla G. et al., 1991). We also demonstrated that oxidation of low density lipoprotein can be highly catalyzed by free heme facilitating modification of plasma apolipoprotein B-100 and the end products of this process lead to vascular endothelial cell injury (Balla G. et al., 1991; Miller and Shaklai, 1994; Li et al., 2006). The origin of the extracellular free heme might be red blood cells (RBCs) often captured in death zones of intraplaque hemorrhage. Plaque lipids of complicated lesions interacts with RBCs causing hemolysis resulting in more free heme inside of the vessel wall (Nagy et al., 2010). During the catalytic process, the porphyrin ring opens, degrades, and iron is liberated leading to the accumulation of footprints of heme catalyzed oxidative reactions including oxidized lipids and proteins, as well as cellular responses and injuries (Balla et al., 2007; Nagy et al., 2010; Jeney et al., 2014).

The importance of protection against heme-catalyzed cell injury is underlined by the existence of natural heme binding proteins, which protect molecules, cells, and the whole organism from the deleterious effects of free heme. Hemopexin (Hpx) binds heme with a stoichiometry of one to one molar ratio with one of the highest known affinities (Kd 10^-13^ M) (Hrkal et al., 1974). Internalization of Hpx-heme complex takes place in part via the scavenger receptor, LDL receptor-related protein1/CD91, although not exclusively (Hrkal et al., 1974; Herz and Strickland, 2001; Hvidberg et al., 2005; Smith and McCulloh, 2015). After intravenous injection of heme-Hpx complexes in rats, uptake was predominantly by hepatocytes (Smith and Morgan, 1978, 1979). It is well established that in plasma and other biological fluids binding of heme by Hpx prevents heme catalyzed oxidative reactions such as LDL oxidation and endothelial cell death (Gutteridge and Smith, 1988; Vincent et al., 1988; Balla G. et al., 1991). Alpha-1-microglobulin (A1M), a radical scavenger and reductase in the plasma and extravascular tissues, is also an effective binder of heme (Kd 10^-6^ M) (Larsson et al., 2004; Åkerström et al., 2007; Åkerström and Gram, 2014). A1M binds heme in plasma, extravascular fluids and cells. A1M binds heme with two binding sites, with a relatively weak one and a stronger, covalent interaction (Larsson et al., 2004; Allhorn et al., 2005). Several reports described the *in vitro* protective effects of A1M in cell cultures against hemoglobin-, heme-, and ROS-induced cell- and tissue damage (Olsson et al., 2008, 2011). Because these two heme binding proteins, A1M and Hpx, protect cells and biological molecules from heme toxicity, they have been proposed as therapeutic agents in pathophysiological conditions where free heme is present; and this has been established in several studies with cell and animal models of human diseases (Schaer et al., 2013, 2014; Vinchi et al., 2016).

The nature of the lethal cellular injury provoked by uptake of “free” heme, *i.e.*, non-protein bound heme is not fully understood. At the present time, the main form of cell death is generally considered to be necrosis caused by heme sensitization to ROS. Heme has been reported to induce ROS in a variety of cell types such as small intestine epithelial cells, neutrophils, macrophages and human umbilical vein endothelial cells provoking various pathophysiological conditions such as intestinal mucosa dysfunction, necrosis, and inflammation (Porto et al., 2007; Fortes et al., 2012; Barcellos-de-Souza et al., 2013; Erdei et al., 2018). One of the major targets of ROS are proteins leading to excessive protein modifications with often detrimental consequences on protein structure and function (Berlett and Stadtman, 1997; Davies, 2016). Because raising intracellular heme can generate ROS resulting in loss off protein functions and fragmentations (Aft and Mueller, 1984; Vincent et al., 1988; Alvarado et al., 2015), we consider that heme-induced ROS triggers endoplasmic reticulum stress (ER stress) together with unfolded protein response (UPR) through ROS-mediated protein damage. We hypothesize that these protein modifications might trigger ER stress and UPR. If protein folding is inhibited or the demand for folding exceeds the folding capacity of the ER, the pathways of UPR are activated leading to ER stress (Schröder and Kaufman, 2005; Walter and Ron, 2011). Evidence is accumulating for an association between vascular diseases and ER stress as well as between atherosclerosis and oxidative stress (Ivanova and Orekhov, 2016; Hong et al., 2017; Kattoor et al., 2017; Yang et al., 2017). However, to date there is no evidences for the role of ER stress in plaque progression. Furthermore, heme has not previously been studied as a trigger of ER stress/UPR in vascular research.

During ER stress, at least three distinct arms of the UPR are initiated, pancreatic ER kinase-like ER kinase (PERK), activating transcription factor-6 (ATF6), and inositol-requiring enzyme 1 (IRE1), all of which are activated by GRP78 (glucose-regulated protein 78 kDa) (Walter and Ron, 2011). Early activation of PERK/phosphorylated eIF2α/ATF4 pathway reduces the protein load of the ER and enables cells to recover from this form of stress (Hamanaka et al., 2005; Raven and Koromilas, 2008). ATF4 initiates the transcription of genes involved in the functional UPR, including those linked to amino acid metabolism, redox homeostasis, and even ER stress-induced apoptosis (Harding et al., 2003; Ameri and Harris, 2008). Prolonged or severe ER stress leads to cell death mediated by CHOP, which is predominantly activated by the PERK/phosphorylated eIF2/ATF4 pathway (Zinszner et al., 1998). The second major player of ER stress is IRE1a, a proximal sensor of ER stress, splices X-box binding protein (XBP1) mRNA by an unconventional mechanism using its endoribonuclease activity (Tirasophon et al., 1998; Lee et al., 2002). Canonical targets of spliced XBP1 include ER chaperones and components of endoplasmic reticulum-associated degradation (ERAD), which eliminate unfolded or misfolded proteins (Acosta-Alvear et al., 2007; He et al., 2010). Prolonged ER stress can also activate apoptosis *via* IRE1-ASK1-JNK pathway (Nishitoh et al., 2002). ATF6 is a transmembrane glycoprotein of ER. Upon ER stress, ATF6 is cleaved and a 50 kDa fragment translocates to the nucleus (Ye et al., 2000; Liu and Kaufman, 2003). ATF6 activates the expression of a number of genes like the ER chaperones including Grp78, Grp94, protein disulfide isomerase, and the components of ERAD and XBP1 (Dorner et al., 1990; Haze et al., 1999; Yoshida et al., 2001; Hirota et al., 2006; Thuerauf et al., 2007; Todd et al., 2008). Overall, these three arms either regulate the expression of numerous genes that restore homeostasis in the ER or may even induce apoptosis (Walter and Ron, 2011).

Endoplasmic reticulum stress was shown to suppress the expression of TGFβ and downstream product collagen type I. TGFβ enhances plaque stability, reduces atherosclerotic plaque size (Bobik, 2006; Chen et al., 2006, 2016; Bot et al., 2009; Reifenberg et al., 2012; Hassan et al., 2018), and is limitedly present in advanced atherosclerotic plaques (Grainger et al., 1995; Bobik et al., 1999; McCaffrey et al., 1999).

The purpose of this study was to investigate whether free heme, in addition to causing intracellular “heme stress” (by raising redox active heme and iron), might also induce ER stress. If so, this would add a new insight into the heme-mediated vessel wall injury in the pathogenesis of atherosclerosis. One of our goals was to demonstrate the close proximity of heme to smooth muscle cells, and the signs of ER stress in these cells in the depth of atherosclerotic plaques in human samples. Using *in vitro* cell culture experiments we mimicked this *in vivo* phenomenon in human aortic smooth muscle cells (HAoSMCs) evaluating heme as a trigger for ER stress using changes in key target proteins of the three arms of the UPR. The final goal was to confirm our second hypothesis, that A1M and Hpx are efficient protectants against this type of complex, toxic heme stress.

## Materials and Methods

### Reagents

Reagents were purchased from Sigma-Aldrich (St. Louis, MO, United States) unless otherwise specified. Hemin chloride stock solution (2 mM) was prepared in sterile 20 mM NaOH on the day of use for each experiment. Human recombinant wild-type A1M was expressed in *E. coli*, purified and refolded as described with an additional ion-exchange chromatography step as outlined (Kwasek et al., 2007; Ahlstedt et al., 2015). Rabbit hemopexin was isolated and purified as previously described (Morgan et al., 1993; Eskew et al., 1999).

### Tissue Samples

Carotid arteries from patients who underwent carotid endarterectomy were obtained from the Department of Surgery at the University of Debrecen. The sample collection was approved by the Scientific and Research Ethics Committee of the Scientific Council of Health of the Hungarian Government under the registration number of DE OEC RKEB/IKEB 3712-2012. Written informed consents were received from the participants. Specimens were examined by trained pathologist and classified according to AHA guidelines. Type I (healthy), IV (atheromatous) and VI (complicated) lesions were selected for further investigation.

### Cell Culture

Human aortic smooth muscle cells were obtained from Cell Applications (San Diego, CA, United States) and Lonza (Allendale, NJ, United States). Cells were grown in low glucose (1g/L) DMEM containing 10% FBS, 100 U/ml penicillin, 100 μg/ml streptomycin and amphotericin B. They were grown to 90% confluence and used from passages 5 to 7. The medium was changed every 2 days. Heme treatments were carried out in serum- and antibiotic-free DMEM. Briefly, hemin chloride stock solution in sterile 20 mM NaOH was diluted in serum- and antibiotic-free DMEM. Cells were washed twice with Hank’s Balanced Salt Solution pH 7.4 supplemented with Ca^2+^ and Mg^2+^ (HBSS + ) and treated with different hemin concentrations for 60 min. Cells were washed with HBSS + and fresh DMEM with 10% fetal bovine serum (FBS) and antibiotics were added and cells were further incubated for 3, 6, and 16 h in a CO2 (5%) incubator. For the A1M and Hpx studies, the proteins were added to hemin diluted in serum- and antibiotic-free DMEM to a concentration of 12.5 μM of A1M (two heme binding sites) and 25 μM of Hpx (one heme binding site), gently mixed then incubated at room-temperature in the dark with gentle agitation for 30 min. Cells treated with 1 μM of thapsigargin, a non-competitive inhibitor of the sarco/endoplasmic reticulum Ca^2+^ATPase (SERCA) as positive controls of ER stress for 3, 6, and 16 h.

For N-acetyl cysteine (NAC), cells were pretreated with NAC (10 mM) in complete growth medium 1 h prior to the heme exposure. Cells were then washed with HBSS+ and treated with NAC (10 mM), heme (25 μM) or with NAC (10 mM) and heme (25 μM) together for 60 min in serum-free DMEM. HAoSMCs were then rinsed with HBSS+ and incubated in complete growth medium alone or supplemented with NAC (10 mM) for 3 h. ER stress markers were then analyzed with q-RT-PCR or immunoblot as described above.

### Cell Lysis and Western Blot

Cells were washed with cold phosphate buffered saline pH 7.4 then lysed with RIPA buffer containing protease and phosphatase inhibitors (50 mM Tris pH 7.5, 150 mM NaCl, 1% Igepal CA-630, 1% Sodium-deoxycholate, 0.1% SDS, 1× Complete Mini Protease Inhibitor Cocktail, 2 × PHOSSTOP phosphatase inhibitor cocktail, and incubated for 15 min on ice. Lysates were clarified by spinning at 16000 × *g*, 4°C for 15 min. Protein content was determined using the bicinchoninic acid assay (Pierce BCA Protein Assay Kit, Thermo Fisher Scientific, Waltham, MA, United States).

Cell extracts (30 μg protein) were electrophoresed on 10% Tris-glycine SDS-PAGE gels, then the proteins were transferred to 0,22 μm PVDF membrane (Bio-Rad, Hercules, CA, United States membrane) and blocked with 5% w/v BSA for 60 min. Primary antibodies against phospho-eIF2α, eIF2α, ATF4, ATF6, XBP1s, and ferritin heavy chain (FTH) from Cell Signaling Technology (Danvers, MA, United States) were diluted 1:1000, while Grp78 and HO1 from Proteintech Group (Manchester M3 3WF, United Kingdom) and diluted 1:5000 in blocking solution. In order to ascertain equivalent protein loading in the samples, the membranes were stripped and reprobed with a mouse anti-human GAPDH antibody (Novus Biologicals, LLC, Littleton, CO, United States) at a dilution of 1:5000. Antigen-antibody complex was detected by WesternBright ECL HRP substrate (Advansta, Menlo Park, CA, United States). Protein bands were quantified with ImageJ software and normalized to GAPDH (Rasband, 1997–2005).

### RNA Isolation and XBP1 Splicing PCR

Cells were grown on six-well plates and total RNA was isolated with Tri Reagent (Zymo Research) and reverse transcribed using High-Capacity cDNA Reverse Transcription Kit (Applied Biosystems Inc., Foster City, CA, United States). XBP1 and GAPDH were amplified with Taq DNA Polymerase (Thermo Fisher Scientific, Waltham, MA, United States). The splice variant of XBP1 was evaluated using the primers and conditions as described by Kosakowska-Cholody et al. (2014). Amplimers were quantified with ImageJ software and normalized to GAPDH.

### Quantitative Reverse Transcription-Polymerase Chain Reaction

Cells were grown on six-well plates and total RNA was isolated and reverse transcribed as described above. ATF4, CHOP, Grp78, and HO1 mRNA expressions were determined by TaqMan Gene Expression Assays (Thermo Fisher Scientific, Waltham, MA, United States) and were normalized to GAPDH (ATF4: Hs00909569_g1; CHOP: Hs00358796_g1; Grp78: Hs00607129_gH; HO1: Hs01110250; GAPDH: Hs02758991_g1; TGFβ: Hs00998133_m1; collagen type I: Hs00164004_m1). Reverse transcriptions and qPCRs were carried out using the C1000 Thermal Cycler with CFX 96 Real Time PCR System (Bio-Rad, Hercules, CA, United States). Relative mRNA expressions were calculated with the ΔΔCt method using GAPDH as internal control.

### Confocal Microscopy

Cells on coverslips were exposed to heme in serum- and antibiotic-free DMEM for 60 min, rinsed twice and then cells were incubated for 3 h in DMEM containing 10% FBS and antibiotics. Cells were fixed in 4% paraformaldehyde in phosphate buffered saline (PBS) pH 7.4 for 15 min. Coverslips were washed with PBS and samples were blocked with 5% goat serum in PBS supplemented with 0.3% Triton X-100 for 60 min. Samples were then incubated with primary antibody against CHOP (Proteintech Group Manchester M3 3WF, United Kingdom) at a 1:500 dilution or against ATF4 (Cell Signaling Technologies, Danvers, MA, United States) at a 1:500 dilution overnight at 4°C in antibody dilution buffer (1% BSA in PBS supplemented with 0.3% Triton X-100). The secondary antibody was a goat anti-rabbit IgG conjugated to Alexa Fluor^®^ 532 (Thermo Fisher Scientific, Waltham, MA, United States) used at a 1:1000 dilution in antibody dilution buffer and incubated for 60 min at room temperature. Nuclei were visualized with Hoechst. Cells treated with 1 μM of thapsigargin for 3 h was used as positive control. Nuclear translocation of CHOP and ATF4 was investigated with TCS SP8 STED microscope using Leica Application Software X (Leica, Mannheim, Germany).

### Detection of Crosslinked Hemoglobin by Western Blot

Detection of crosslinked hemoglobin in three healthy carotid arteries and three complicated carotid lesions by Western blot was performed as described in our previous study using HRP-conjugated goat anti-human Hb polyclonal antibody (ab19362-1 Abcam, Cambridge, United Kingdom) at a dilution of 1:15000 (Nagy et al., 2010).

### Determination of Oxidized Hemoglobin and Heme Content in Healthy Carotid Arteries and Complicated Carotid Lesions

Determination of oxidized hemoglobin concentration in healthy carotid arteries and complicated carotid lesions was performed according to the method of Winterbourne CC (Winterbourn, 1990). Heme measurements in tissue samples were performed as described by Huy et al. (2005).

### Immunohistochemistry

The common carotid artery specimens were fixated with PBS buffered formaldehyde (4%) solution (4%) at pH 7.4 for 1 to 3 day – based on the size of the sample. In case of calciphicated samples 1.0 M/l EDTA/Tris buffer was used for the decalcification after fixation. The vascular segments were embedded in paraffin wax, than 3–5 um thick slides were prepared through deparaffination used by xylene and ethanol. After inhibition of endogenous peroxidase (0.5% for 20 min) activity slides were subjected to antigen retrieval in a buffer solution (pH 9.0, RE7119, Leica, Wetzlar, Germany). For immunohistochemistry, samples were incubated with Dako EnVision FLEX Peroxidase-Blocking Reagent (Dako, Glostrup, Denmark) for 5 min in a wet chamber. Slides were then washed with EnVision^TM^ FLEX Wash Buffer, Tris-buffered saline solution containing Tween 20, pH 7.6 (± 0.1). Antigen retrieval was performed in the epitope retrieval solution (RE-7119, Tris/EDTA–based buffer containing surfactant, Leica, Wetzlar, Germany) at pH 9 using a pressure cooker. Slides were then washed with distilled water and EnVision^TM^ FLEX Wash Buffer, Tris-buffered saline solution containing Tween 20, pH 7.6 (±0.1). Next slides were incubated with CHOP (clone: rabbit polyclonal 15204-1-AP Proteintech Group, Rosemont, IL 60018, United States) primary polyclonal antibody at a dilution of 1:1000 used OPTIVIEW DAB DETECTION KIT based on protocol No. 6396500001. Serial sections next slides were incubated with GRP78/BIP antibody (clone: rabbit polyclonal 11587-1-AP Proteintech Group, Rosemont, IL 60018, United States) primary polyclonal antibody at a dilution of 1:2000 used ULTRAVIEW UNIVERSAL DAB DETECTION KIT based on protocol No. 5269806001. The intensity and distribution of CHOP and GRP78/BIP specific immunostaining was assessed by light microscopy (Leica DM2500 microscope, DFC 420 camera and Leica Application Suite V3 software, Leica). Counterstain with Gill Hematoxylin solution (105175 Merck Millipore, Billerica, MA, United States). Rinse in running tap water for 2–5 min. Dehydrate through 95% ethanol for 1 min, 100% ethanol for 2 × 3 min. Clear in xylene for 2 × 5 min. The slices were coverslip with mounting medium.

### Determination of ROS

Reactive oxygen species generated by heme was measured using the chloromethyl derivative of 2′,7′-dichlorodihydrofluorescein diacetate (CM-H2DCFDA; Life Technologies, Carlsbad, CA, United States). Cells were seeded in a clear- bottom black 96-well plate overnight in DMEM medium containing 10% FBS and antibiotics. Cells were washed three times and incubated in HBSS + supplemented with CM-H2DCFDA (10 μM) for 30 min in a CO2 incubator. Cells were washed three times and incubated with corresponding to vehicle control (VC), thapsigargin (1 μM) ER stress positive control (PC) or with various doses of heme (1, 10, and 25 μM) for 60 min. Cells were washed thoroughly, and fluorescence intensity was measured applying 485 nm excitation and 530 nm emission wavelengths. In some experiments, heme (25 μM) was pre-incubated either with hemopexin (25 μM) or recombinant A1M (12.5 μM) in HBSS + for 30 min before the treatments. To mitigate oxidative stress, ROS was scavenged by the addition of NAC (10 mM) to the experimental medium before and during the treatments. Briefly, cells were pre-incubated with ROS scavenger NAC (10 mM) for 60 min in HBSS+ then exposed to heme (25 μM) together with NAC (10 mM).

### Cell Viability Assay

Cell viability was determined by the MTT assay. Briefly, cells were cultured and treated in 96-well plates for the indicated time. Then cells were washed with PBS, and 100 μL of 3-[4,5-dimethylthiazol-2-yl]-2,5-diphenyl-tetrazolium bromide (0.5 mg/mL) solution in HBSS was added. After a 90-min incubation, the MTT solution was removed, formazan crystals were dissolved in 100 μL of DMSO and optical density was measured at 570 nm.

### Statistical Analysis

Data are shown as mean ± SD. Statistical analysis was performed by one-way ANOVA test followed by Bonferroni correction or unpaired *t*-test. A value of *p* < 0.05 was considered significant.

## Results

### The ER Chaperone Grp78 and Cell Death Marker CHOP Are Both Upregulated in Hemorrhaged Complicated Lesions Compared to Atheromas and Healthy Carotid Arteries

**FIGURE 1:**
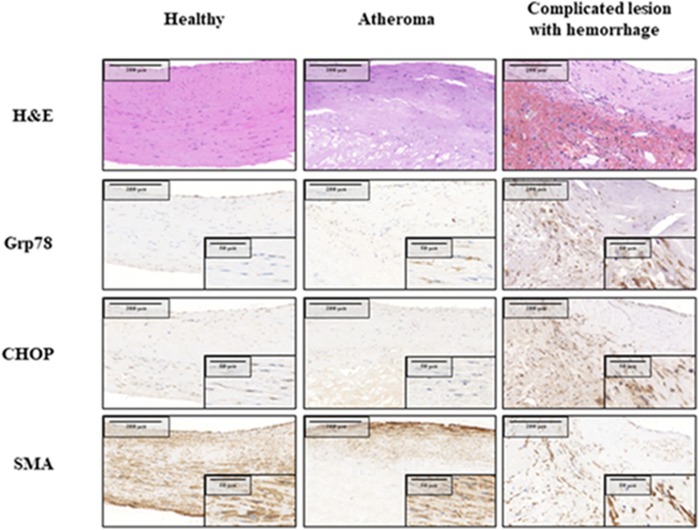
Expression of ER chaperone Grp78 and death signal protein CHOP in healthy carotid wall, atheromatous plaque and complicated lesion with hemorrhage. Immunomorphological features associated with different stages of atherosclerotic process: serial sections of healthy carotis wall **(Left)**, atheromatous plaque **(Middle)** and complicated (hemorrhagic) lesion **(Right column)** are shown. H&E staining, SMA, CHOP and Grp78 immunohistochemistry are presented from the same tissue areas, all pictures and all inserts with the same magnification.

First, we analyzed whether hemorrhaged complicated lesions show increased ER stress marker expression compared to healthy carotid arteries or atheromas (Figure 1). Macroscopic features and hematoxylin-eosin-stained slides covered the full spectrum of atherosclerotic lesions within the collected carotid endarterectomy specimens. Healthy vessels, atheromas and complicated, freshly hemorrhagic lesions are shown. The selected samples were stained for smooth muscle actin, Grp78 and CHOP using immunohistochemistry. Normal appearing carotid wall tissue showed weak level of Grp78 and CHOP expression, mostly located to the nucleolar and perinucleolar regions. Simple (stabile) atheromas displayed separated layers of smooth muscle cell bunches surrounding the lipid deposition. CHOP and Grp78 protein expression in these cells was nearly the same compared with the healthy control carotid samples. Ruptured, hemorrhagic complicated lesions are presented with highly increased CHOP and Grp78 immunopositivity appearing in both the cytoplasm and the nucleolus of the polygonal activated smooth muscle cells in the interstitium of the artery.

### Oxidation of Hemoglobin Occurs in Complicated Lesions Resulting in Accumulation of Free Heme

**FIGURE 2:**
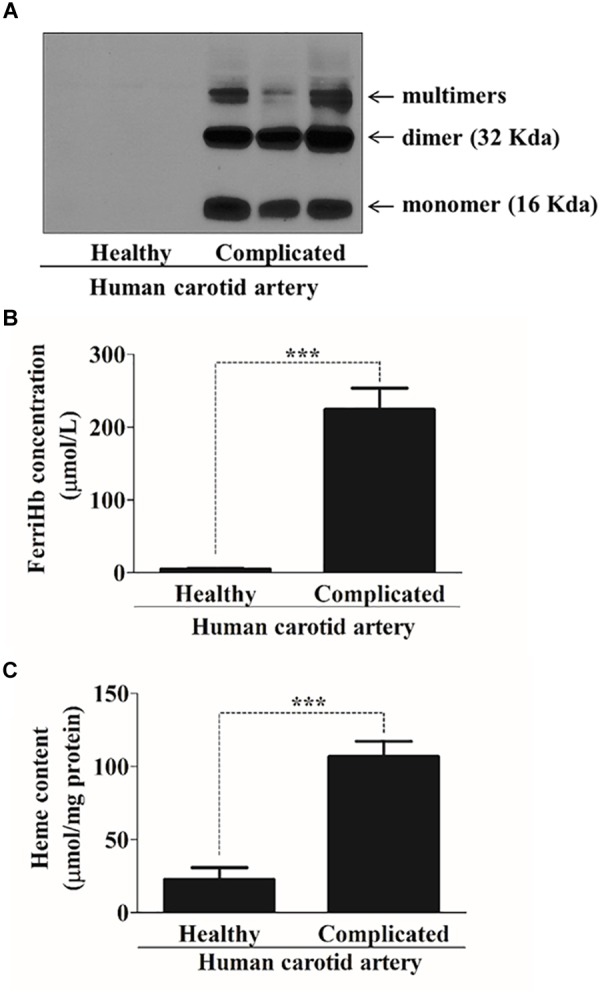
Complicated lesions contain oxidized forms of hemoglobin and are characterized by high heme content. **(A)** Representative Western blot (one out of five performed) showing Hb expression in three human healthy and complicated carotid artery (20 μg/lane). **(B)** A spectral scan of human healthy (*N* = 17) and complicated (*N* = 11) carotid artery tissue lysates were taken and concentrations of ferriHb (μmol/L) were calculated based on the visible spectra. Values were normalized to protein content of the tissue lysates. ^∗∗∗^*p* < 0.0001 using unpaired *t*-test. **(C)** Total heme concentrations (μmol/mg protein) of human healthy (*n* = 14) and complicated (*n* = 11) carotid artery tissue lysates were determined spectrophotometrically and calculated according to the heme standard curve. ^∗∗∗^*p* < 0.0001 using unpaired *t-*test.

Our previous studies revealed that complicated lesions with hemorrhage contained oxidized forms of hemoglobin (Nagy et al., 2010). Here, we also demonstrate that in specimens of such atherosclerotic plaques, the hemoglobin was oxidized (methemoglobin containing ferric heme) as reflected by the accumulation of cross-linked dimers, tetramers and multimers (Figure 2A). As expected, methemoglobin readily released its heme moieties (Figures 2B,C). Since cells are exposed to heme in the complicated hemorrhaged atherosclerotic plaques, we next addressed whether ER stress in vascular smooth muscle cells was provoked by heme.

### Heme Activates the PERK/eIF2/ATF4/CHOP Arm of ER Stress Sensors

**FIGURE 3:**
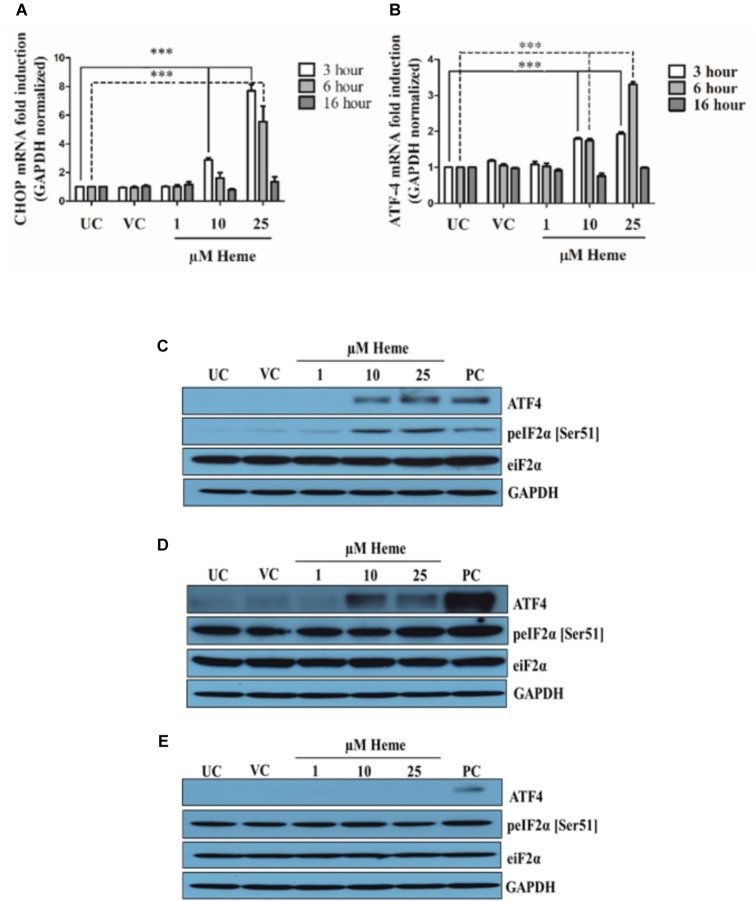
Heme activates the PERK/ATF4/CHOP arm of ER stress in a time- and dose-dependent manner. HAoSMCs were treated with various doses of heme (1,10, and 25 μM) or corresponding vehicle solution to highest heme dose (25 μM) in serum-free DMEM for 60 min, then medium was changed to DMEM with 10% FCS and antibiotics. ER stress markers were measured after 3, 6, or 16 h. Thapsigargin (1 μM) treated cells were used as positive control **(A–E)**. **(A,B)** Relative expressions of CHOP **(A)** and ATF4 **(B)** mRNA levels were determined by qRT-PCR, normalized to GAPDH and compared to the untreated controls at each time points. UC, untreated control; VC, vehicle control; PC, positive control, thapsigargin treated. Results are presented as mean ± SD of five independent experiments performed in duplicates. ^∗∗∗^*p* < 0.001. **(C–E)** Representative Western blots of whole cell lysates from five independent experiments are shown representing eIF-2 phosphorylation and ATF4 protein levels **(C)** 3, **(D)** 6, and **(E)** 16 h after the heme treatment. UC, untreated cells; VC, vehicle control cells; PC, positive control cells.

CHOP is the terminal effector protein of the PERK pathway and our first aim was to investigate its expression in HAoSMCs in response to heme. CHOP mRNA was measured in extracts of cells made 3, 6, and 16 h after exposure to heme (1, 10, and 25 μM). Thapsigargin-treated (1 μM) cells were used as positive controls. As shown in Figure 3A, heme induced CHOP mRNA expression in a time- and dose-dependent manner. Significant enhancement was observed after HAoSMCs were exposed to heme (10 and 25 μM) 3 h after the treatment and declined there after back to the control level 16 h after heme treatment. Thapsigargin treated cells showed more robust CHOP activation compared to heme even after 16 h of incubation where heme induced CHOP levels declined to the control level (Supplementary Figure 1). The expression of CHOP at the protein level together with its nuclear translocation were also assessed with immunofluorescence 3, 6, and 16 h after heme exposure. Untreated cells as well as vehicle controls had very low fluorescence as did the cells exposed to low levels of heme (1 μM). Cells treated either with 10 or 25 μM of heme showed increased fluorescence signal consistent with higher expression, and, furthermore, nuclear translocation of CHOP 3 h after the heme exposure demonstrating ER stress (Supplementary Figure 2). Fluorescence signals in cells treated with 10 μM of heme markedly declined thereafter within the next 3 h similar to the controls, while levels remained elevated for up to 16 h in the cells exposed to 25 μM (Supplementary Figures 3, 4). CHOP is predominantly up-regulated *via* the PERK-eIF2α-ATF4 signaling branch of the UPR; therefore, to gain a deeper insight into the mechanism of CHOP activation in response to heme, we analyzed eIF2α phosphorylation as well as ATF4 expression. These two proteins are the upstream regulatory element of CHOP. Heme at 10 and 25 μM, but not 1 μM, transiently induced the phosphorylation of eIF2α (p-eIF2α) and subsequently ATF4 expression too, both at the mRNA and protein levels (Figures 3B–E). As with the induction of CHOP mRNA, eIF2α phosphorylation as well as ATF4 mRNA expression showed a dose- and time dependency. Phosphorylation of eIF2α was significantly increased 3 h after heme treatment and was only detectable 6 h after the treatment when the highest heme doses were applied (25 μM; Figures 3C,D). eIF2α phosphorylation as well as ATF4 expression declined to basal levels 16 h after the heme challenge (Figure 3E). ATF4 mRNA levels showed slightly different kinetics from CHOP mRNA expression with a peak at 3 h after 10 μM heme treatment and remained constant up to 6 h. ATF4 levels increased gradually up to 6 h after the treatment when HAoSMCs were exposed to 25 μM heme (Figure 3B). Accordingly, the ATF4 was translocated to the nucleus in cells incubated with heme (10 and 25 μM) further supporting activation of the PERK pathway within 3 h after the heme exposure (Supplementary Figure 5).

### Heme Also Activates the IRE1/XBP1s Arm of ER Stress Sensors in a Time- and Dose-Dependent Manner

**FIGURE 4:**
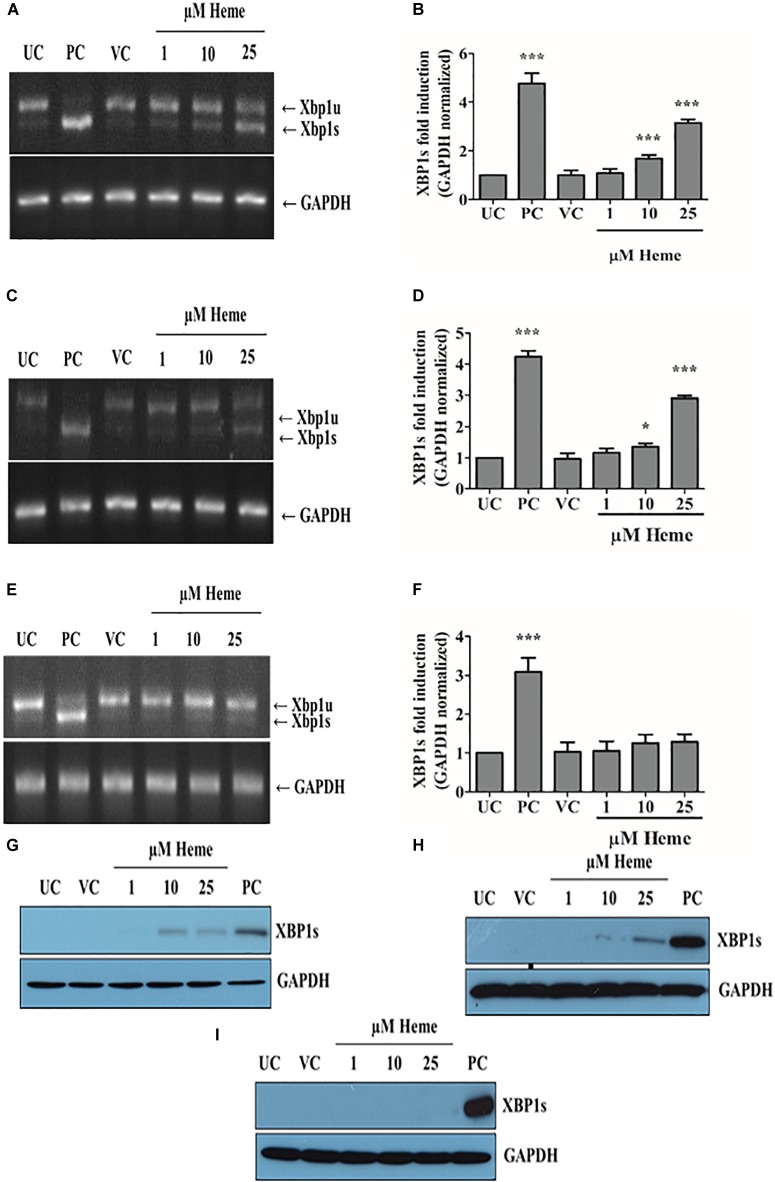
Heme activates the IRE1/XBP1 arm of ER stress in a time- and dose-dependent manner. Expression of spliced XBP1 (XBP1s) was measured with RT-PCR followed by densitometric analysis of XBP1s after running PCR products on 2% agarose gel. XBP1s levels were normalized to GAPDH. **(A,B)** XBP1s levels measured 3, **(C,D)** 6, and **(E,F)** 16 h after the heme challenge. Agarose gels **A,C,E** show one representative image of five independent experiments performed in duplicates. Graphs **B,D,F** are presented as mean ± SD of five independent experiments performed in duplicates. ^∗^*p* < 0.05, ^∗∗∗^*p* < 0.001 compared to untreated cells. **(G–I)** Representative immunoblots of five independent experiments showing XBP1s expression **(A)** 3, **(B)** 6, and **(C)** 16 h after the heme exposure. UC: untreated control; VC: vehicle control; PC: positive control, thapsigargin treated cells.

Upon activation of the UPR, IRE1 IRE1α splices XBP1 mRNA resulting XBP1s protein, which is a potent transcriptional activator that induces expression of many UPR responsive genes. To examine whether heme activates this arm of the classical ER stress in addition to the PERK arm, we measured XBP1 splicing catalyzed by activated IRE1 after challenging the HAoSMCs with different concentrations of heme. As previously, control cells were treated with 1 μM of thapsigargin. The PCR results showed that heme (10 and 25 μM) induced the activation of the canonical IRE1/XBP1s pathway in a time- and concentration-dependent manner, which was demonstrated by a transient increase in the ratio of XBP1 spliced (XBP1s) and XBP1 unspliced (XBP1u) levels (Figures 4A–F). IRE1/XBP1 activation was the most pronounced 3 h after heme exposure (not shown). The level of XBP1s mRNA markedly decreased 6 h after the treatment when HAoSMCs were exposed to 10 μM of heme, but remained induced when cells were treated with 25 μM of heme (Figures 4C,D). XBP1s levels decreased to the control level 16 h after the treatment (Figures 4E,F). Neither vehicle nor low dose heme (1 μM) activated IRE1/XBP1 pathway (Figures 4A–F) consistent with our data on the PERK pathway. The protein expression of the spliced form of XBP1 correlated well with the mRNA expression and showed time- and dose-dependence (Figures 4G–I). These findings suggest that the IRE1 pathway was activated by a certain level of heme but transiently, in contrast to the sustained activation of the PERK pathway.

### Heme Also Activates the ATF6 Arm of ER Stress in Time- and Dose-Dependent Manner

**FIGURE 5:**
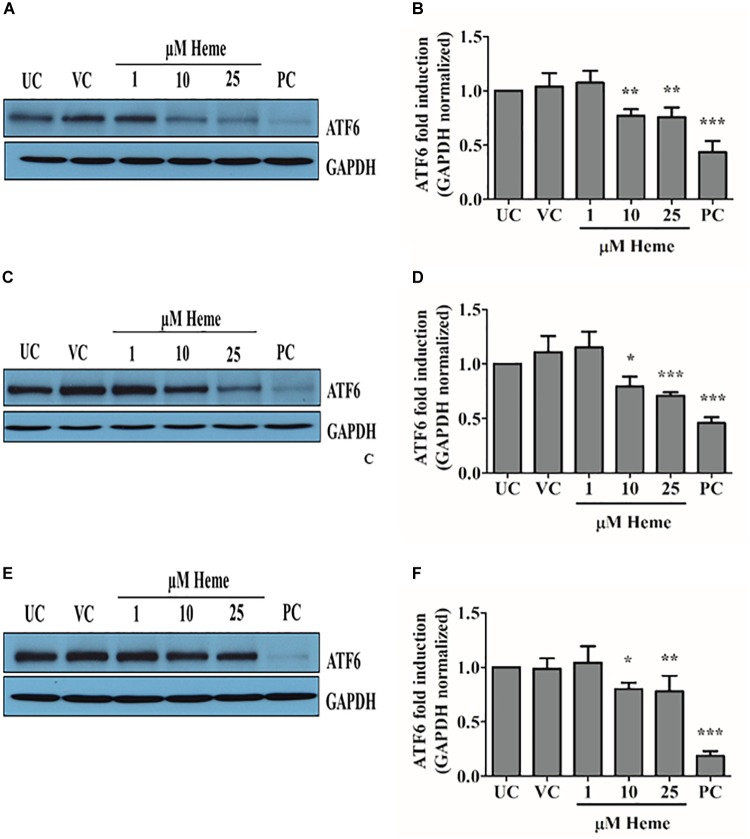
Heme activates ATF6 arm of ER stress in a time- and dose-dependent manner. Proteolytic activation of ATF6 was investigated using Western blot and quantified by densitometry **(A–F)**. **(A,B)** ATF6 levels measured 3, **(C,D)** 6, and **(E,F)** 16 h after the heme challenge. Western blots **(A,C,E)** show one representative image of five independent experiments. Graphs **(B,D,F)** are presented as mean ± SD of five independent experiments performed in duplicates. ^∗^*p* < 0.05, ^∗∗^*p* < 0.01, ^∗∗∗^*p* < 0.001 compared to untreated cells.

ATF6 is a membrane bound transcription factor that activates genes in response to ER stress because as unfolded proteins accumulate. ATF6 is cleaved and its cytoplasmic domain enters the nucleus, where it induces a set of chaperones (Grp78, Grp94, calreticulin) that are involved in stress adaptation and survival. To investigate whether heme induces ATF6-mediated stress adaptive response, we assessed the extent of proteolytic activation of ATF6 by immunoblotting. Heme at concentrations of 10 and 25 μM, but not at 1 μM, induced a dose-dependent proteolytic activation of ATF6 (Figures 5A–F) causing a time- and dose-dependent decrease in ATF6 protein levels. Noticeably, the ATF6 pathway was still activated 16 h after heme challenge indicating a robust and long-lived response (Figures 5E,F). Thus, the adaptive ATF6 pathway is also activated in response to heme.

### Heme Increases the Expression of the Grp78, an ER Chaperone

**FIGURE 6:**
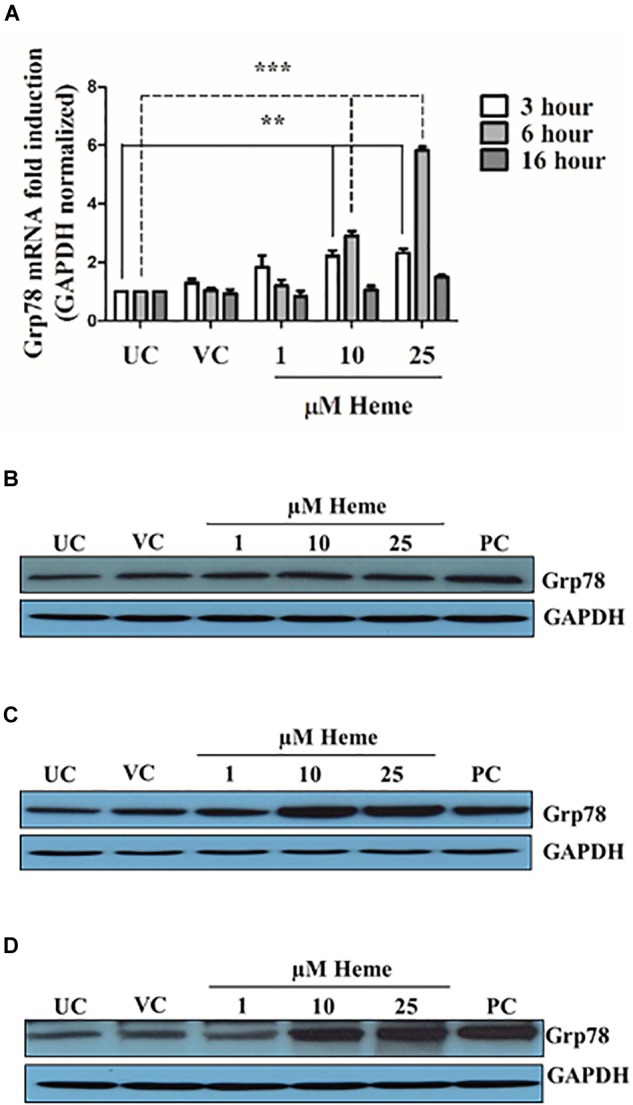
Heme induces Grp78 expression in a time- and dose-dependent manner. Cells were treated either with heme or vehicle as mentioned above. Grp78 expression was measured using qRT-PCR and immunoblot **(A–D)**. **(A)** Relative expressions of Grp78 was determined by qRT-PCR, normalized to GAPDH and compared to the untreated controls at each time points. Results are presented as mean ± SD of five independent experiments performed in duplicates. ^∗∗^*p* < 0.01, ^∗∗∗^*p* < 0.001. **(B–D)** Representative Western blots of whole cell lysates from five independent experiments are shown representing Grp78 protein levels **(B)** 3, **(C)** 6, and **(D)** 16 h after the heme treatment.

The trafficking of ATF6 from the ER to the Golgi for processing by site 1 and site 2 proteases is controlled by the ER chaperone BiP/Grp78. Thus, this protein is considered the master regulator of ER stress as well as a remarkable chaperone that is itself up-regulated during ER stress. Therefore, we next examined whether or not Grp78 responded to heme. Heme treatment increased the expression of Grp78 mRNA 3 h after heme exposure (Figure 6A). Importantly, a trend in Grp78 mRNA induction was apparent even in response to low dose heme (1 μM) within 3 h (Figure 6A). Grp78 mRNA levels gradually rose up to 6 h after treatment when HAoSMCs were exposed to either 10 or 25 μM of heme (Figure 6A). Grp78 mRNA levels returned to control levels after 16 h unless cells had been treated with 25 μM heme in which case Grp78 protein remained significantly increased (*p* ≤ 0.05). Accordingly, Grp78 protein levels were considerably up-regulated when cells were treated with heme (10 and 25 μM) compared to control cells (Figures 6B–D). Notably, Grp78 protein levels for heme treatments and vehicle controls did not differ 3 h after the heme exposure showing an induction phase of stress adaptation (Figure 6B). Parallel with Grp78 mRNA induction, a robust increase in Grp78 protein levels were detected 6 and 16 h after HAoSMCs were exposed to either 10 or 25 μM of heme (Figures 6B–D). Our results support that the ER chaperone Grp78 responds to heme stress. Thapsigargin-treated cells showed a more robust induction Grp78 (data no shown).

### Heme Markedly Increase Heme Oxygenase 1 (HO1) and Ferritin Heavy Chain Expression in HAoSMCs

**FIGURE 7:**
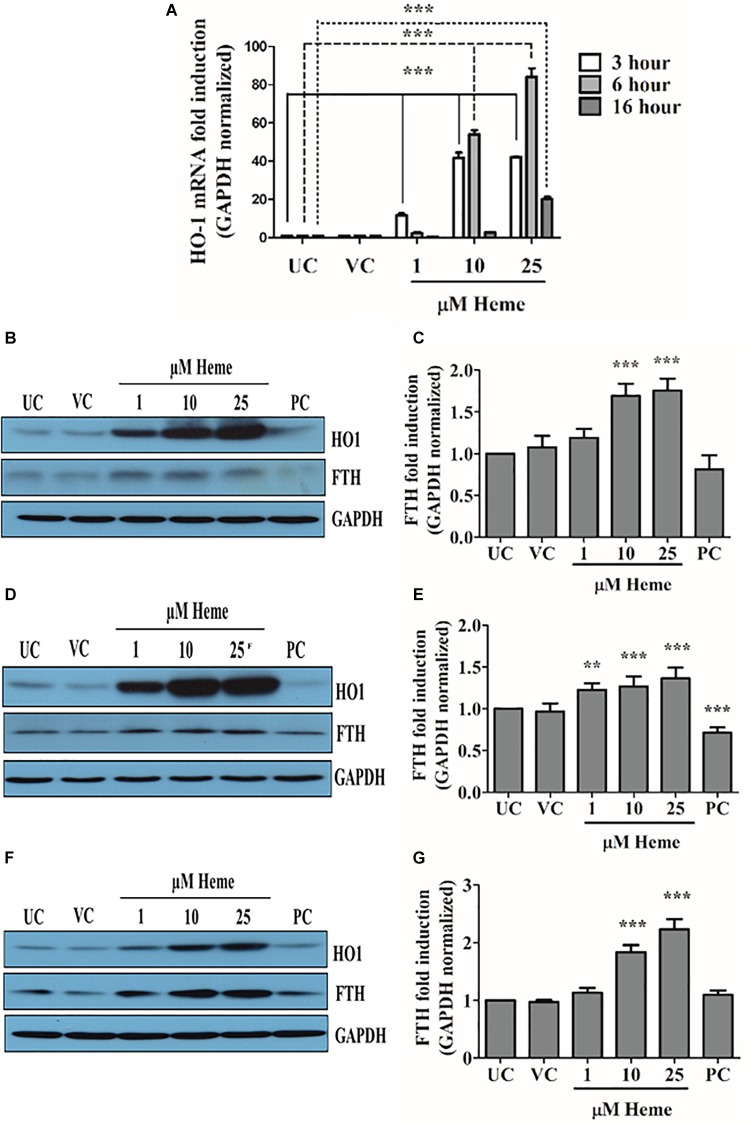
Heme induces marked HO1 and ferritin heavy chain expression in a concentration- and time-dependent manner. Cells were treated either with heme or vehicle as mentioned above. HO1 expression was measured using qRT-PCR and immunoblot, while ferritin heavy chain by immunoblot **(A–G)**. **(A)** Relative expressions of HO1 was determined by qRT-PCR, normalized to GAPDH and compared to the untreated controls at each time points. Results are presented as mean ± SD of five independent experiments performed in duplicates. ^∗∗∗^*p* < 0.001. **(B,D,F)** Representative Western blots of whole cell lysates from five independent experiments are shown representing HO1 and H-ferritin (FTH) protein levels **(B)** 3, **(D)** 6, and **(E)** 16 h after the heme treatment. **(C,E,G)** Densitometric analysis of FTH expression **(C)** 3, **(E)** 6, and **(G)** 16 h after the heme exposure. Results are presented as mean ± SD of five independent experiments. FTH expressions are normalized to GAPDH and compared to the untreated controls at each time points. UC, untreated control; VC, vehicle control; PC, positive control, thapsigargin treated. ^∗∗^*p* < 0.01, ^∗∗∗^*p* < 0.001.

To confirm and monitor the intracellular effect of heme and its catabolism, we assessed the expression of HO1 and the translation of ferritin heavy chain. As expected, heme markedly increased HO1 mRNA as well as protein levels and also the iron release from heme catabolism by HO1 induced ferritin heavy chain levels (Figures 7A–G). The extent of HO1 induction is highly correlated with intracellular heme levels in a concentration-dependent fashion, and was apparent in response to 1 μM heme. HO1 and ferritin heavy chain were still elevated 16 h after heme treatment indicating high levels of HO1 and ferritin heavy chain.

### The Heme Binding Proteins A1M and Hpx Attenuate the Activation of PERK, IRE1/XBP1, and ATF6 Pathways as Well as Grp78 Induction Protecting Against Heme Induced ER Stress

**FIGURE 8:**
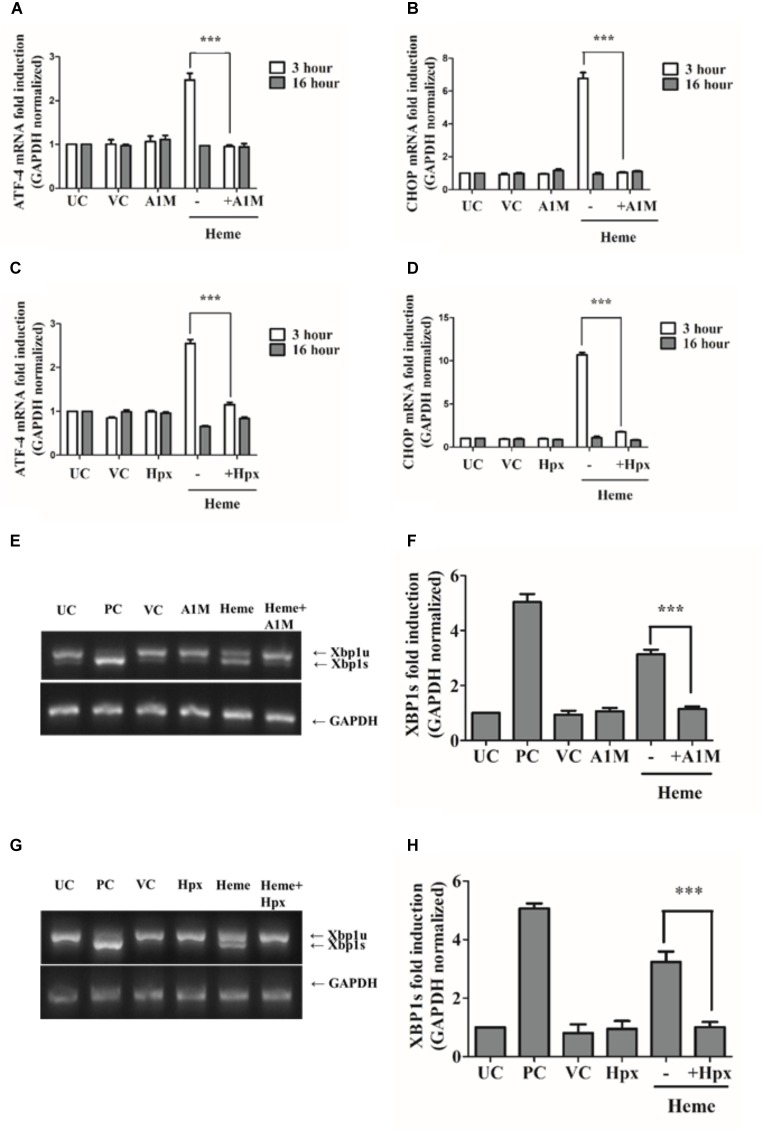
A1M and Hpx attenuates ATF4 and CHOP gene induction as well as IRE1/XBP1s activation. Cells were treated with heme (25 μM) or heme + rA1M (25 μM heme + 12.5 μM rA1M) or equimolar amonts of heme + Hpx (25 μM heme + 25 μM Hpx) for 60 min in serum-free DMEM. Medium was then replaced with DMEM + 10% FCS and antibiotics. ATF4 and CHOP gene induction in response to heme were measured using qRT-PCR 3 and 16 h after the heme challenge **(A–D)**, while XBP1 splicing was assessed 3 h after the heme treatment by RT-PCR followed agarose gel electrophoresis and quantified by densitometry **(E–H)**. **(A–B)** Relative expressions of ATF4 and CHOP in response to heme and heme + rA1M complex were determined by qRT-PCR, normalized to GAPDH. ATF4 and CHOP levels of rA1M + heme treated cells were compared to those detected in heme challenged cells. Results are presented as mean ± SD of five independent experiments performed in duplicates. ^∗∗∗^*p* < 0.0001 using unpaired *t*-test **(C–D)** Relative expressions of ATF4 and CHOP in response to heme and heme + Hpx complex were determined by qRT-PCR, normalized to GAPDH. ATF4 and CHOP levels of Hpx + heme treted cells were compared to those detected in heme challenged cells. Results are presented as mean ± SD of five independent experiments performed in duplicates. ^∗∗∗^*p* < 0.0001 using unpaired *t*-test. **(E–F)** Cells were treated with heme or heme + rA1M as described above. Expression of spliced XBP1 (XBP1s) was measured 3 h after the treatment with RT-PCR followed by densitometric analysis of XBP1s after running PCR products on 2% agarose gel. XBP1s levels were normalized to GAPDH. Results are presented as mean ± SD of five independent experiments performed in duplicates. ^∗∗∗^*p* < 0.0001 using unpaired *t*-test. **(G,H)** Cells were treated with heme or heme + Hpx as described above. Expression of spliced XBP1 (XBP1s) was measured 3 h after the treatment with RT-PCR followed by densitometric analysis of XBP1s after running PCR products on 2% agarose gel. XBP1s levels were normalized to GAPDH. Results are presented as mean ± SD of five independent experiments. ^∗∗∗^*p* < 0.0001 using unpaired *t*-test.

**FIGURE 9:**
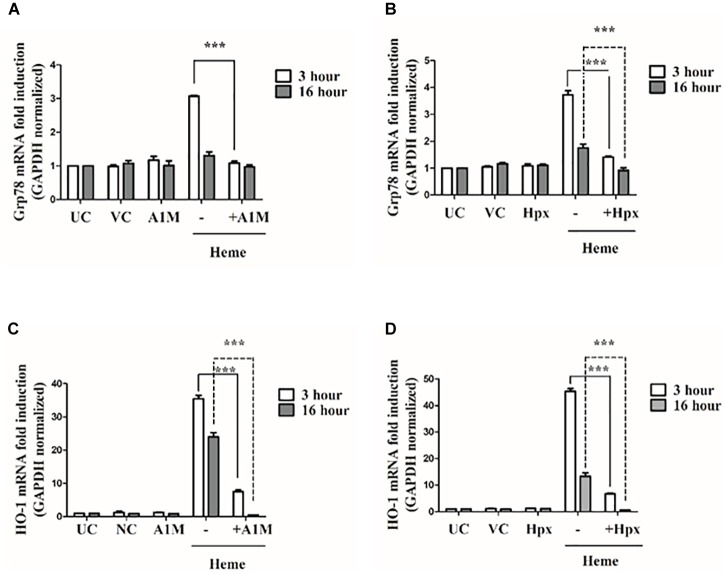
A1M and Hpx attenuates Grp78 and HO1 gene induction. Cells were treated with heme or heme + rA1M or equimolar amonts of heme + Hpx as described in the legend to Figure 8. Grp78 and HO1 expression was measured using qRT-PCR 3 and 16 h after the treatment **(A–D)**. **(A)** Relative expressions of Grp78 in response to heme and heme + rA1M complex were determined by qRT-PCR, normalized to GAPDH. Grp78 levels of rA1M + heme treated cells were compared to those detected in heme challenged cells. Results are presented as mean ± SD of five independent experiments performed in duplicates. ^∗∗∗^*p* < 0.0001 using unpaired *t*-test **(B)** Relative expressions of Grp78 in response to heme and heme + Hpx complex were determined by qRT-PCR, normalized to GAPDH. Grp78 levels of Hpx + heme treted cells were compared to those detected in heme challenged cells. Results are presented as mean ± SD of five independent experiments performed in duplicates. ^∗∗∗^*p* < 0.0001 using unpaired *t*-test. **(C)** Relative expressions of HO1 in response to heme and heme + rA1M complex were determined by qRT-PCR, normalized to GAPDH. HO1 levels of rA1M + heme treated cells were compared to those detected in heme challenged cells. Results are presented as mean ± SD of five independent experiments performed in duplicates. ^∗∗∗^*p* < 0.0001 using unpaired *t*-test. **(D)** Relative expressions of HO1 in response to heme and heme + Hpx complex were determined by qRT-PCR, normalized to GAPDH. HO1 levels of Hpx + heme treted cells were compared to those detected in heme challenged cells. Results are presented as mean ± SD of five independent experiments. ^∗∗∗^*p* < 0.0001 using unpaired *t*-test.

A1M and Hpx are proteins that protect cells from heme toxicity by acting as extracellular anti-oxidants, sequestering heme and limiting its chemical reactivity. Our next question was whether recombinant A1M (rA1M) and Hpx inhibit the ER stress and UPR pathways induced by heme. For these experiments HAoSMCs were incubated with heme-protein complexes produced by incubating heme (25 μM) with either 12.5 μM of rA1M (a stoichiometric molar ratio of heme: protein of 2:1) or 25 μM of Hpx (a stoichiometric molar ratio of heme: protein of 1:1). We analyzed ATF4, CHOP, Grp78 and HO1 mRNA expression as well as IRE1-mediated XBP1 splicing 3 or 16 h after challenging cells with either heme or the heme-protein complexes. When presented as heme-protein complexes, both rA1M and Hpx completely attenuated ATF4, CHOP, and Grp78 mRNA expression as well as inhibited XBP1 splicing 3 h after the treatment (Figures 8A–H, 9A,B) and markedly decreased HO1 mRNA expression at 3 h (Figures 9C,D).

**FIGURE 10:**
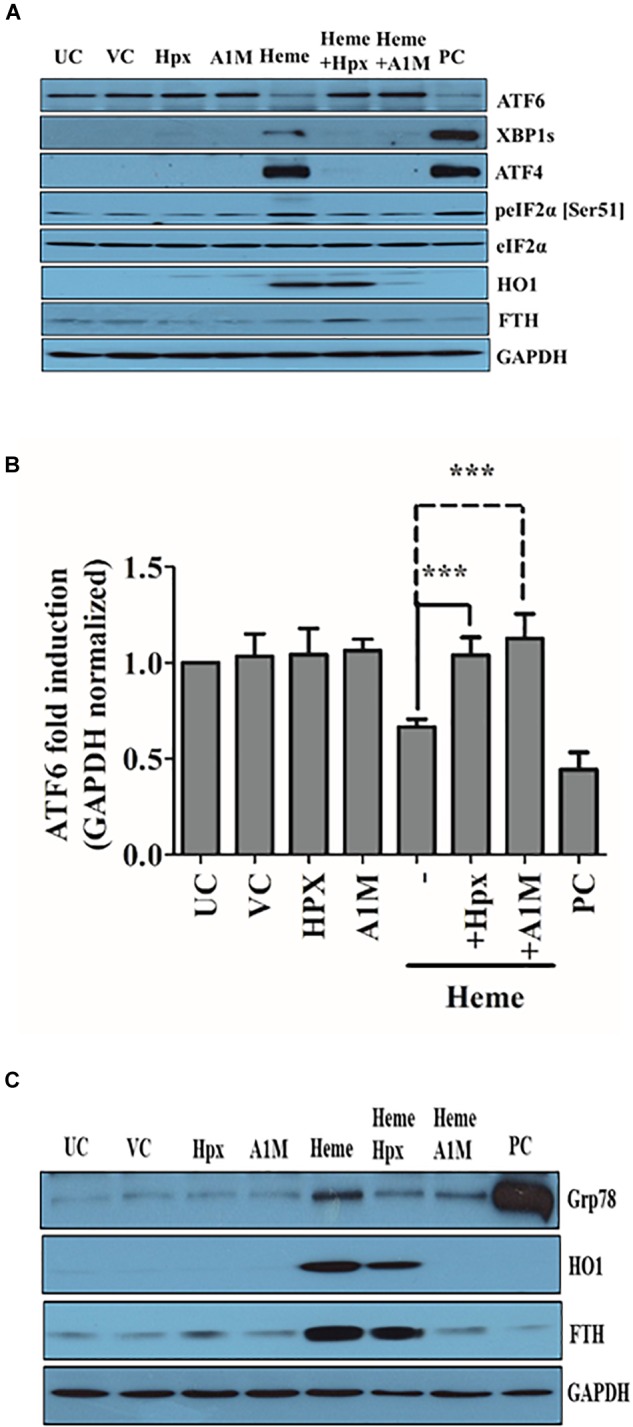
A1M and Hpx attenuates the activation of PERK and ATF6 pathways. Cells were treated with heme or heme + rA1M or equimolar amonts of heme + Hpx as described above. Early ER stress markers (phosphorylated eIF2α, ATF4, and ATF6) were measured with immunoblot, while CHOP was assessed by immunofluorescence 3 h after the treatments **(A–C)**. Heme stress marker HO1 and FTH were measured 3 and 16 h after the treatment. **(A)** Representative Western blots of whole cell lysates from five independent experiments are shown representing ATF6 proteolysis, eIF2α phosphorylation and subsequent ATF4 induction as well as HO1 and FTH expression. **(A)** Analysis of CHOP expression and nuclear translocation in response to heme and heme + rA1M or heme + Hpx treatment. **(B)** Graph shows ATF6 proteolysis induced by heme or heme + rA1M or heme + Hpx assessed by densitometric analysis of ATF6 immunoblots. Results are presented as mean ± SD of five independent experiments performed in duplicates. ^∗∗∗^*p* < 0.0001 using unpaired *t*-test. **(C)** Representative Western blots of whole cell lysates from five independent experiments are shown representing Grp78, HO1, and FTH inductions 16 h after the treatment.

Both rA1M and Hpx prevented the phosphorylation of two early markers of ER stress eIF2a and ATF4 at the protein level (Figure 10A). Accordingly, rA1M and Hpx completely inhibited the expression and nuclear translocation of CHOP and proteolysis of ATF6 as demonstrated by immunofluorescence staining of CHOP and densitometric analysis of ATF6 immunoblots, respectively (Figures 10A,B and Supplementary Figure 6). Furthermore, rA1M as well as Hpx markedly reduced the expression of late ER stress marker Grp78 as shown in Figure 10C. At the protein level, when rA1M was in the medium, HO1 and ferritin did not appear to be induced. Surprisingly, the levels HO1 and ferritin heavy chain protein were increased when Hpx was present in the medium. This contrasts with our previous data on endothelial cells, when Hpx prevented HO1 induction (Balla G. et al., 1991). In an earlier *in vivo* work, heme-Hpx is targeted to the liver after intravenous injection in rats (Smith and Morgan, 1979). The scavenger receptor termed low density related lipoprotein receptor (LRP1), that binds heme-Hpx complexes very tightly (Kd ∼4 nM, Hvidberg et al., 2005) *in vitro*, has been reported to be expressed on vascular smooth muscle cells (Okada et al., 1996; Swertfeger et al., 2002; Lillis et al., 2005). Thus, LRP1 on these HAoSMCs may account for some uptake of heme-HPX or, under these experimental conditions, some heme was not bound to Hpx and entered the cells.

Overall, these data show that both rA1M and Hpx attenuate the activation of all ER stress pathways provoked by heme.

### N-Acetyl Cysteine Attenuates ROS Generation and ER Stress in Heme-Exposed Cells

**FIGURE 11:**
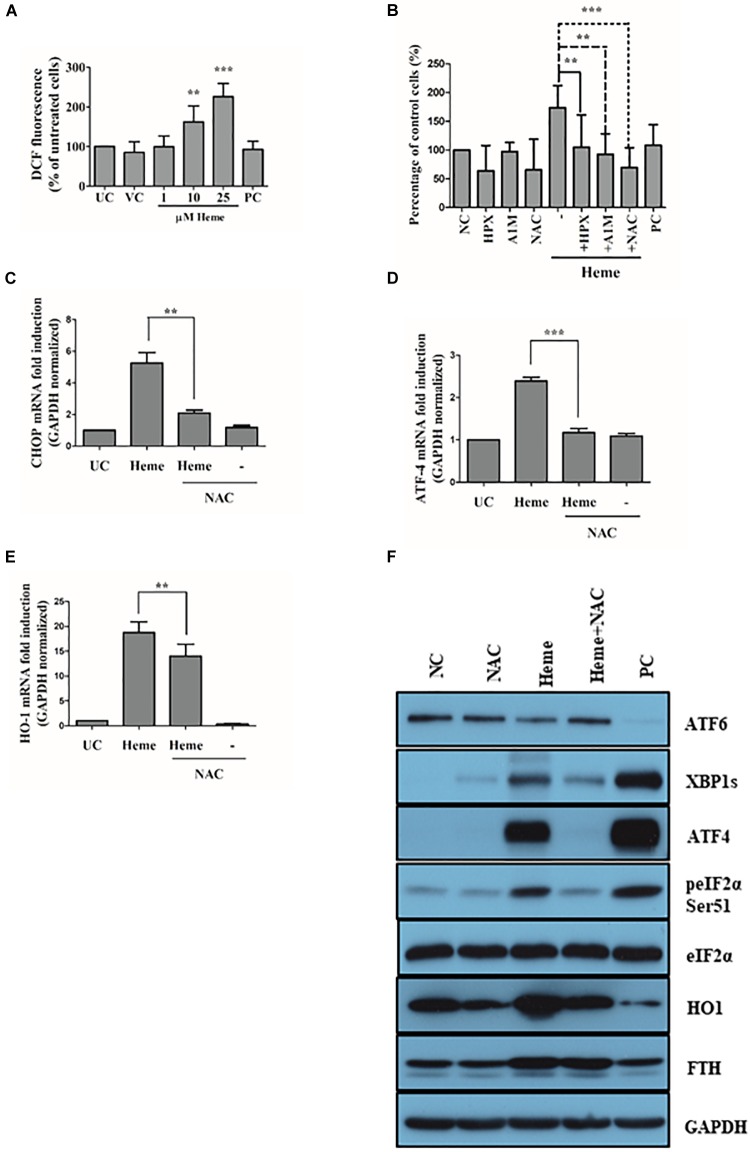
Heme-generated ROS is involved in heme-induced endoplasmic reticulum (ER) stress. Cells were treated with heme (25 μM) alone or heme (25 μM) + rA1M (12.5 μM) or heme (25 μM) + Hpx (25 μM) or heme (25 μM) + N-acetyl cysteine (NAC; 10 mM) as described for Figure 8 followed by the analysis of ROS generation and ER stress marker expression **(A–F)**. **(A)** Heme induced ROS generation was assessed by CM-H2DCFDA assay. Results are presented as mean ± SD of five independent experiments. ^∗∗∗^*p* < 0.0001. **(B)** Inhibition of heme-induced ROS generation by Hpx, rA1M and NAC using CM-H2DCFDA assay. Results are presented as mean ± SD of five independent experiments. ^∗∗∗^*p* < 0.0001 **(C–E)** Relative expressions of CHOP, ATF4 and HO1 in response to heme and heme + NAC were determined by qRT-PCR, normalized to GAPDH. mRNA levels of heme + NAC treted cells were compared to those detected in heme challenged cells. Results are presented as mean ± SD of five independent experiments performed in duplicates. ^∗∗∗^*p* < 0.0001 using unpaired *t*-test. **(F)** Representative Western blots of whole cell lysates from five independent experiments are shown representing ATF6 proteolysis, XBP1s expression, eIF2α phosphorylation and subsequent ATF4 induction as well as HO1 and FTH expression.

It was previously described that heme induces ROS in a number of cell types (Porto et al., 2007; Fortes et al., 2012; Barcellos-de-Souza et al., 2013; Erdei et al., 2018). Therefore, we tested whether heme triggers ROS generation in HAoSMCs (Figures 11A–F). As expected, heme (10 and 25 μM) induced ROS in a dose-dependent manner measured by the CM-H2DCFDA assay (Figure 11A). Importantly, heme-induced ROS generation was effectively inhibited by either the ROS scavenger NAC or the extracellular heme binding protein hemopexin and A1M (Figure 11B). Due to its potent antioxidant properties, NAC was reported to attenuate ROS-mediated ER stress in a number of pathologies (Porto et al., 2007; Fortes et al., 2012; Barcellos-de-Souza et al., 2013; Erdei et al., 2018). Thus, we investigated whether NAC could prevent heme-induced ER stress as well. Our results showed that NAC inhibited ATF4 mRNA expression, significantly but not completely prevented heme-provoked elevation of CHOP mRNA, and decreased the expression of HO1 (Figures 11C–E). The ER stress moderating effect of NAC was pronounced at protein level as well, since NAC inhibited the heme-induced activation of ER stress pathways analyzed by immunoblot (Figure 11F). In addition, NAC decreased HO1 protein levels in heme-treated cells as well and was able to significantly, but not completely reduce the expression and nuclear translocation of CHOP by heme (Supplementary Figure 7). These data suggest that heme induced ROS generation can be effectively prevented either by capturing heme in the extracellular space by rA1M or Hpx or by the antioxidant NAC. By lowering ROS generation, NAC was able to inhibit heme-induced ER stress as well.

### Determination of Cell Viability of HAoSMCs in Response to Heme

Next, we analyzed whether heme induced ER stress triggers cell death in HAoSMCs. Cells were treated with various doses of heme (1, 10, and 25 μM, respectively) and cell viability was tested by MTT assay (Supplementary Figure 8). Thapsigargin (1 μM) treated cells were used as positive control for ER stress. Our data showed that neither heme nor the ER stressor thapsigargin induced cell death in our experimental conditions.

### Heme Decreases the Expression of Transforming Growth Factor β (TGFβ) and Collagen Type I in HAoSMCs

**FIGURE 12:**
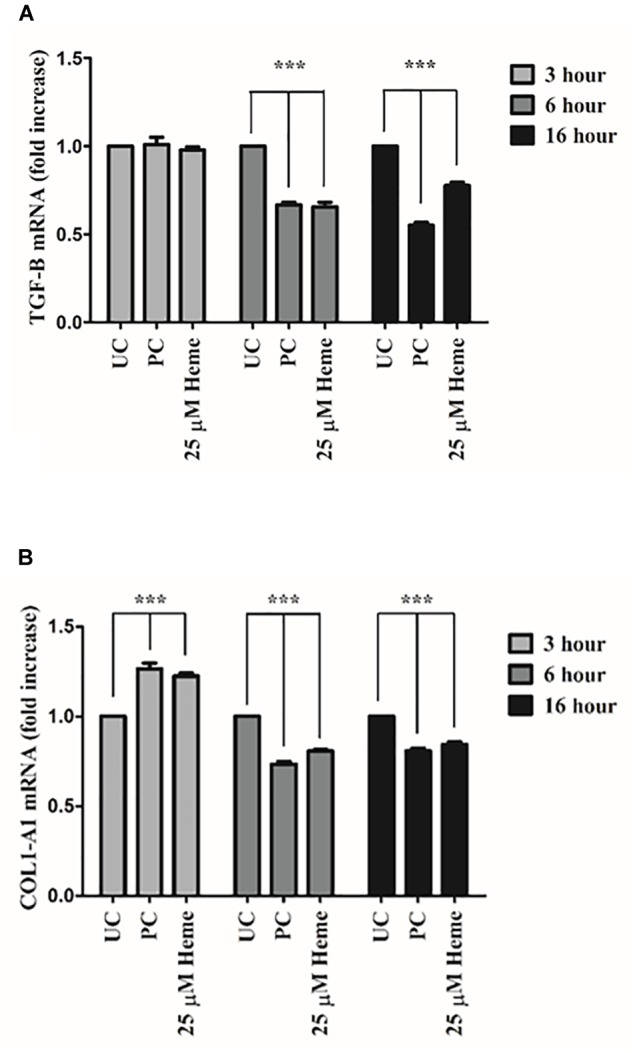
Heme attenuates TGFβ and collagen type I expression in HAoSMCs. Cells were treated with heme (25 μM) or thapsigargin (1 μM) as described for Figure 3 and TGFβ and collagen type I mRNAs were quantified by qRT-PCR **(A,B)**. Relative expressions of TGFβ **(A)** and collagen type I **(B)** mRNA levels were determined by qRT-PCR, normalized to GAPDH and compared to the untreated controls at each time points. UC: untreated control; PC: positive control, thapsigargin treated. Graphs **(A,B)** are presented as mean ± SD of five independent experiments performed in duplicates. ^∗∗∗^*p* < 0.001 compared to untreated cells.

TGFβ and its downstream product collagen type I plays a pivotal role in the maintenance and repair of the protective fibrous cap in atherosclerotic plaques. Therefore, we next investigated whether heme induced ER stress affects TGFβ and subsequent collagen type I expression. Cells were treated with heme (25 μM) or thapsigargin (1 μM) then TGFβ and collagen type I mRNA expressions were quantified *via* qPCR 3, 6, and 16 h after the treatments. Our results showed that heme (25 μM) as well as thapsigargin (1 μM) significantly inhibited both TGFβ and collagen type I mRNA expressions at 6 and 16 h after treatments (Figures 12A,B). Both thapsigargin and heme increased collagen type I mRNA expression at 3 h after heme or thapsigargin challenge (Figure 12B). These data suggest that heme effect on fibrotic gene expression are biphasic, and both heme and thapsigargin markedly reduce fibrotic gene expression after an initial induction phase.

## Discussion

There is increasing evidence for a link between vascular diseases and ER stress as well as between atherosclerosis and oxidative stress (Ivanova and Orekhov, 2016; Hong et al., 2017; Kattoor et al., 2017; Yang et al., 2017). Several reports have demonstrated that ER stress and UPR are involved in cardiovascular diseases and multiple risk factors of atherosclerosis such as free cholesterol, 7-ketocholesterol, phospholipolyzed LDL or oxidized LDL have been described to trigger ER stress in macrophages, vascular endothelial cells, and HAoSMCs (Pedruzzi et al., 2004; Kedi et al., 2009; Sanson et al., 2009; Gora et al., 2010; Yao et al., 2010; Chistiakov et al., 2014).

The purpose of our research was to assess how heme, a known risk factor for atherosclerosis, contributes to the pathology of this complex and prevalent cardiovascular disease.

Heme is an amphipathic iron-protoporphyrin IX molecule that is, in part, responsible for human pathologies including atherosclerosis (Andrade et al., 2010; Larsen et al., 2010; Nagy et al., 2010; Nath and Katusic, 2012; Frimat et al., 2013; Elphinstone et al., 2016). It was previously reported that heme may mediate the oxidative degradation of proteins (Aft and Mueller, 1984; Vincent, 1989). A recent study published by our research group showed that the protein titin, responsible for the passive elasticity of muscle and in the contraction of striated muscle tissue, is sensitive to heme driven oxidation, can be involved in the increase of the passive force of myocardium driven by heme triggered oxidative protein modifications (Alvarado et al., 2015). Heme, which is also capable of sensitizing vascular endothelial cells toward ROS, and moreover, catalyzes the oxidative modification of LDL to oxLDL, represents an important risk factor of vessel wall injury (Balla G. et al., 1991; Jeney et al., 2002). Great number of observations confirmed the presence of ROS injuries in atherosclerotic plaques, and intriguingly, heme together with cell-free heme proteins are also present in human vascular wall samples (Jeney et al., 2002, 2014; Balla et al., 2005; Nagy et al., 2010). Hemoglobin interacts with lipid particles triggering further lipoprotein modifications and hemoglobin oxidation. The main hemoglobin derivative is methemoglobin readily capable to release its heme moieties to the surrounding extracellular space (Nagy et al., 2010; Jeney et al., 2014).

The focus of this study was to explore the role of heme in ER stress in HAoSMCs, since these cells are central players of vascular diseases. They mainly exist in a contractile phenotype regulating vascular tone and express a variety of HAoSMC-specific contractile markers, including α-smooth muscle action (SMA) and calponin (Owens, 1995). However, in response to injury, HAoSMCs can undergo a phenotypic switch that is characterized by reduced contractile marker expression and increased proliferation (Rensen et al., 2007). This conversion plays a pathophysiologic role in the development of atherosclerosis (Rzucidlo et al., 2007). However and alternatively, it might be beneficial during atherogenesis to compensate for the loss of HAoSMC due to apoptosis and subsequent inflammation (Bennett et al., 2016). Thus, a considerable body of evidence suggests that HAoSMCs are key players in vascular diseases, which makes them an ideal target to examine the potential role of heme-induced ER stress in atherosclerosis.

Here, we show for the first time that heme induces ER stress and the UPR in HAoSMCs. The different arms of ER stress were followed after heme treatment in a series of cell culture experiments. Our results support that heme activated all three ER stress responsive branches: PERK-eIF2α, IRE1α-XBP1, and ATF6 pathways. Specifically, HAoSMC cultures responded to heme by the activation of ATF6 and IRE1α-XBP1 pathways as well as through an increase in eIF2α phosphorylation and subsequent ATF4 induction *via* the PERK pathway. Increased levels of Grp78 as well as ATF6 activation in response to heme suggest a triggering of the adaptive phases of the cellular stress response. No cytotoxicity was detected in response to heme via MTT assay.

Phosphorylation of eiF2α and ATF4 expression in response to heme possibly indicate that heme triggered oxidative stress together with ER stress in HAOSMCs. ATF4 is a Janus faced factor, since ATF4-mediated integrated stress response, initiated by eIF2α phosphorylation, has been shown to protect cells against oxidative stress (Harding et al., 2003). These observations suggest that ATF4, a marker of ER stress and UPR, might be directly involved in the heme stress response. These results support our hypothesis that heme-induced ROS and oxidative stress are involved in heme-induced ER stress. Heme was reported to enhance the generation of ROS in a variety of cell types which might be involved in heme-related pathologies (Porto et al., 2007; Fortes et al., 2012; Barcellos-de-Souza et al., 2013; Erdei et al., 2018). One of the major targets of ROS are proteins leading to excessive protein modifications with often detrimental consequences for protein structure and hence function (Berlett and Stadtman, 1997; Ehrenshaft et al., 2015; Davies, 2016). We heve demonstrated that heme does induce ROS formation in a concentration-dependent manner in HAoSMCs. Furthermore, by decreasing the ROS level, NAC markedly inhibited heme-induced ER stress in HAoSMCs showing that ROS formation contributed to ER stress triggered by heme.

ATF4 subsequently activates CHOP, a pro-apoptotic protein (Oyadomari and Mori, 2004). Our results on CHOP induction in response to heme correlates well with studies reporting activation of PERK/eIF2α/ATF4/CHOP pathway in a response to oxidized LDL, which, like heme, is considered a pathological factor of atherosclerosis (Hong et al., 2014; Tao et al., 2016). In addition, a strongly positive correlations between CHOP expression and the progression of atherosclerosis in human coronary arteries and carotid lesions as well as ApoE^-/-^ mice have been reported during the past few years (Myoishi et al., 2007; Thorp et al., 2009; Tsukano et al., 2010). Several lines of evidence suggested that IRE1/XBP1 activation are likely involved in vascular diseases (Zeng et al., 2009; Liberman et al., 2011). We observed after HAoSMCs were incubated with heme that the IRE1/XBP1 pathway was activated, but only transiently. We propose that XBP1 activation might act as a pro-survival signal for HAoSMCs through the activation of ER-associated degradation (ERAD) to reduce misfolded protein loads. In addition, the adaptive ER stress response involves initiation of the ATF6 arm, which induces a number of chaperones such as Grp78, Grp94, and calreticulin (Okada et al., 2002). One potential beneficial effect of ATF6 activation is supported by a recent study showing that ATF6 decreases myocardial ischemia/reperfusion damage (Jin et al., 2017). The sustained activation of ATF6 pathway observed in response to heme suggests that ATF6 might facilitate the recovery from ER stress. Grp78, the master regulator of ER stress, is a pro-survival factor and a representative chaperone of adaptive ER stress response (Rutkowski et al., 2006). Under oxidative stress Grp78 could be protective (Yu et al., 1999; Jiang et al., 2017). Here, we revealed that heme induces the sustained and robust up-regulation of Grp78. Therefore, our data support that Grp78 together with other chaperones, possibly induced by ATF6, could potentially contribute to cell survival during heme-induced ER stress, depending upon the levels of heme.

The protective HO1/ferritin system is induced for heme catabolism that releases Fe(II) in cells in response to heme and hemoglobin (Balla J. et al., 1991; Vercellotti et al., 1994). Thus, as expected we detected a robust induction of HO1 and ferritin in HAoSMCs after incubation with heme. Significantly, our data show that high levels of heme can occur in localized hemolytic conditions in atherosclerosis. ER stress develops in spite of the normally protective response of proteins involved in heme trafficking to HO1 in the ER. Intriguingly, several lines of evidence support that there is a direct interaction between HO1, the key player of heme stress, and PERK, a representative kinase of canonical in ER stress. In the cytosol, Nrf2, which is a key transcriptional activator of the HO1 gene, is a direct substrate of PERK. Thus, upon ER stress Nrf2 is released from Keap1 in the cytosol and phosphorylated by PERK. Phospho-Nrf2 then translocates into the nucleus where it acts to induce a number of stress responsive genes in addition to HO1, such as glutathione S-transferase and the rate limiting enzyme in glutathione biosynthesis, glutamylcysteine synthetase (Venugopal and Jaiswal, 1996; Wild et al., 1999; Nguyen et al., 2000; Cullinan et al., 2003; Loboda et al., 2016). Activation of this Nrf2 pathway is independent of eIF2α phosphorylation and promotes cell survival upon, and also after, ER stress (Cullinan et al., 2003; Cullinan and Diehl, 2004). These findings demonstrate that PERK initiates two parallel ways to cope with ER stress, one of which (HO1/ferritin) is also at least part of the traditional pathway of heme stress. Interestingly, there are multiple links between Keap1/Nrf2/HO1 and PERK pathways, since Nrf2 and the downstream protein of PERK arm, ATF4, share certain gene targets (Thimmulappa et al., 2002; Kwak et al., 2003; Lee et al., 2003) including CHOP, a key regulator of ER stress. Nrf2 inhibits CHOP expression, whereas ATF4 promotes it (Harding et al., 2000; Ma et al., 2002; Cullinan and Diehl, 2004). Certain regulatory effects may be cell type specific. Zong and collaborators demonstrated that Nrf2 affected CHOP expression by modulating the binding of ATF4 to the CHOP promoter in thyroid cancer cells, and the accumulation of Nrf2 negatively regulated CHOP induction (Zong et al., 2012). These authors concluded that Nrf2 might inhibit the binding of ATF4 to CHOP promoter.

**FIGURE 13:**
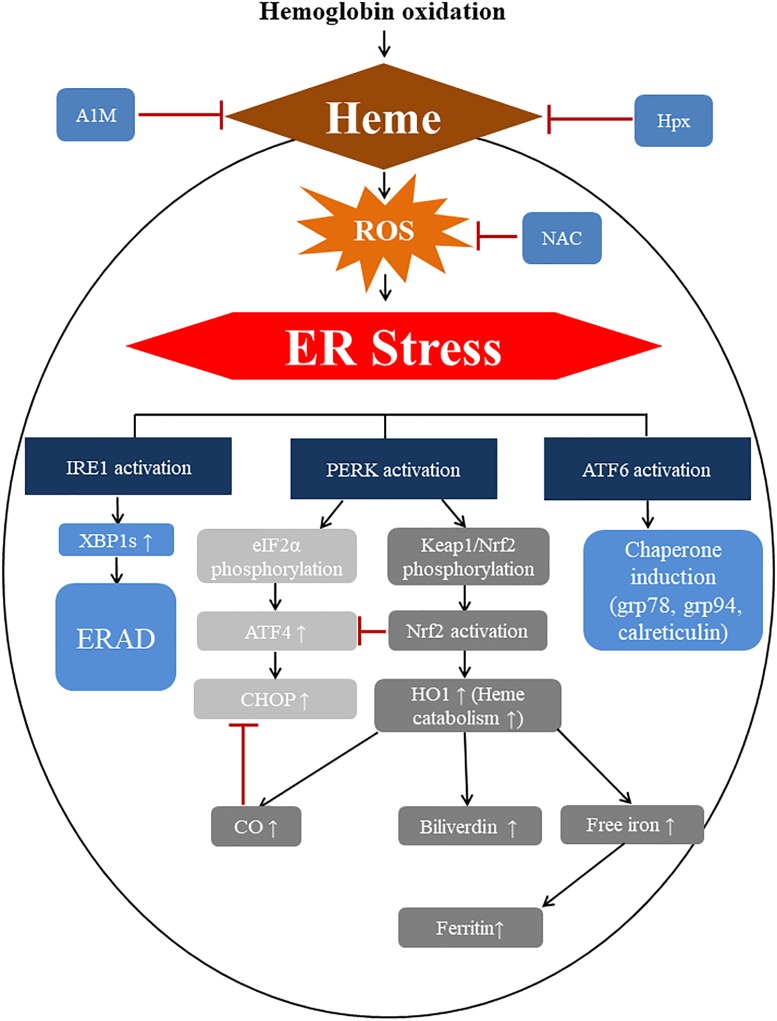
Mechanism of heme induced ER stress with possible therapeutic interventions. Heme released from oxidized hemoglobin enters into cells across the plasma membrane (by unknown mechanisms) possibly *via* passive diffusion or transporters (remains to be defined) and induces ROS intracellularly with subsequent protein oxidative damage that leads PERK activation, phosphorylation of eIF2α with subsequent ATF4 as well as CHOP induction and nuclear translocation. Oxidation of Keap1 releases Nrf2, which PERK is known to phosphorylate Keap1/Nrf2 followed by nuclear translocation of Nrf2, which interacts with various targets and possibly influences CHOP expression directly as well as indirectly. For example, Nrf2 might directly inhibit ATF4 binding to CHOP promoter thereby inhibiting ATF4-mediated CHOP expression. Nrf2 also induces HO1, one of the key heme responsive genes. HO1 decreases the heme burden of cells and releases heme catabolites such as carbon-monoxide (CO), biliverdin (which is converted to the antioxidant bilirubin by biliverdin reductase in the cytosol), and iron (Fe^2+^). CO might inhibit CHOP expression, which suggests that Nrf2 may negatively influence CHOP in an indirect, HO1-mediated way as well. Activation of XBP1s through IRE1 in response to heme possibly induces ER-associated protein degradation (ERAD), which decreases misfolded protein loads and reduces unfolded protein burden of ER. Heme-induced activation of ATF6 also facilitates chaperone expression like Grp78 increasing the folding capacity of ER. Thus, heme potentially activates both pro-apoptotic and pro-survival ER responses, however, our data support that the pro-apoptotic response is only transitory, while pro-survival ones (ATF6, Grp78) are permanently activated. The observed marked induction of heme responsive genes as well as ER stress responsive genes suggest that these pathways interact with each other to cope with heme-induced stress. Finally, capturing heme in the extracellular space by the heme binding proteins alpha-1-microglobulin and hemopexin or decreasing reactive oxygen species level by NAC attenuate heme-induced ER stress.

Another possible interaction between the effects of raising intracellular heme levels/heme stress and ER stress is the key enzyme that lowers intracellular heme, HO1, which catalyzes heme degradation to carbon monoxide, biliverdin (which is converted to bilirubin) and free Fe(II) iron. Liu et al. (2005) suggested that HO1 may inhibit CHOP transcription by modulating the level of carbon monoxide. HO1 is known to be induced during ER stress triggered by various perturbants such as brefeldin A, thapsigargin, and homocysteine. Apoptosis provoked by these stressors/stresses was potentiated by HO1 inhibition, which shows that HO1 contributes to cell survival during ER stress (Liu et al., 2005). Significantly, these published data support a mechanism whereby heme-induced ER stress can rescue cells from CHOP-mediated cells death. This would take place by a direct inhibition of ATF4 binding to CHOP promoter and by an HO1-related carbon monoxide mediated way. Our current understanding on HIER stress in summarized in Figure 13.

There are extracellular protective agents against heme-induced ER stress. For example, the potential therapeutic efficacy of the heme binding protein Hpx against heme toxicity has been shown in patients (Janz et al., 2013; Schaer et al., 2013; Jung et al., 2015). Also, there are animal models of various human diseases such as severe sepsis as well as hemolytic mice models where heme mediated damages are pronounced (Larsen et al., 2010; Vinchi et al., 2013; Smith and McCulloh, 2015). Moreover, Hpx prevents macrophage inflammatory activation which can contribute to chronic tissue injury in hemolytic disorders (Vinchi et al., 2016). In our present study Hpx completely prevented ER stress induced by heme. Also, the protective effect of Hpx against heme-catalyzed cytotoxicity in human and bovine endothelial cells was previously demonstrated by us when heme uptake and heme potentiated H_2_O_2_- and polymorphonuclear leukocyte-mediated cytotoxicity were completely blocked when Hpx was added simultaneously and levels that stoichiometrically bound heme (Balla G. et al., 1991). Here, we found that Hpx acting as an extracellular antioxidant significantly inhibited heme induced ER stress but not HO1/ferritin expressions, which may be due in part to expression of the heme-Hpx binding protein LRP1 The cytoprotective effect of Hpx is reinforced by a study on neuronal cells, where free heme decreased cell survival, while heme complexed with Hpx did not trigger cell death even at supraphysiological concentrations (Li et al., 2009). After endocytosis, heme-Hpx complexes induce HO1 expression in mouse hepatoma, in HL60 cells as well as in mouse primary neurons, where data showed that HO1 expression is required for Hpx-mediated cytoprotection (Alam and Smith, 1989; Alam et al., 1999). *In vivo* studies in rats implicated the liver as the main organ for uptake of heme-hemopexin complexes (Smith and Morgan, 1978). However, it is worth mention that the Hpx bound heme in phosphate buffered saline (PBS pH 7.4) *in vitro* to ∼ 100% after addition of 1 molar equivalent of stock mesoheme in DMSO. The efficiency of heme binding generally the same for both proto- and meso-heme. Thus, it is unexpected to see HO1 and ferritin induction that would indicate and excess of heme in the mixture in the cell experimental medium of heme and Hpx. Because the Hpx preparation was determined to bind heme in PBS *in vitro* to ∼ 100% after addition of 1 molar equivalent of stock mesoheme in DMSO to 1 molar equivalent of Hpx, the potential free heme in the experimental medium causing unexpected HO1 and ferritin expression is attributed to the experimental medium and conditions which are different from the *in vitro* efficacy of heme binding potential of Hpx. On the other hand, HAoSMCs are thought to express the scavenger receptor LRP1 that binds heme-Hpx (Okada et al., 1996; Swertfeger et al., 2002; Hvidberg et al., 2005; Lillis et al., 2005) so some endocytosis of heme-Hpx may have taken place, leading to the unexpected HO1 and H-Ferritin expression. Moreover, it might be beneficial that Hpx allowed the activation of the endogenous, protective HO1/ferritin heavy chain system to cope with further stress stimuli. In our present research, we have also shown that A1M inhibited heme induced ER stress. Overall, our research further bolster up the feasibility of Hpx-based therapeutics in diverse pathologies in which heme-mediated tissue damage plays an etiopathogenetic role and the potential therapeutic role of A1M in diseases where free heme/Hb is present. In other *in vitro* models, A1M hindered intracellular oxidation and upregulation of HO1 induced by heme in primary keratinocytes also in erythroid cell line, K562 (Olsson et al., 2008, 2011). A1M prevented cell lysis of K562 cells caused by heme and cleared the cells from bound heme. Similar effects have been described in heme-treated skin explants (Olsson et al., 2008, 2011).

We have also confirmed that significant amounts of heme accumulate in complicated atherosclerotic plaques. It has been demonstrated that cell-free Hb is present in ruptured atheromas and exists in oxidized forms. We observed that HAoSMCs, even those at quite a distance from the border of intraplaque bleeding, showed the characteristics of ER stress with marked expression of ER stress/UPR marker Grp78 and CHOP. Therefore, our data underline the importance of a new phenomenon; the presence of unique sanctuary areas in complicated lesions, where excess free hemoglobin and heme most likely interact with cells leading to excessive protein modifications with hallmarks of ER stress and UPR. These data indicate that excess heme present in complicated lesions might trigger ER stress *in vivo*.

A body of evidence suggest that TGFβ promotes plaque stability and reduces atherosclerotic plaque size (Bobik, 2006; Chen et al., 2006, 2016; Bot et al., 2009; Reifenberg et al., 2012; Hassan et al., 2018). In addition, inhibition of TGFβ signaling accelerates atherosclerosis, decreases collagen synthesis and induces hemorrhages and iron deposition (Mallat et al., 2001; Lutgens et al., 2002). Overall, factors that decrease collagen synthesis in HAoSMCs greatly affect their ability to maintain the protective fibrous cap in atherosclerotic lesions. TGFβ signaling is negatively influenced by HO1, the key effector protein of heme catabolism in hepatocellular carcinoma cells (Park et al., 2018). In NIH3T3 and human lung fibroblasts, TGFβ-induced collagen synthesis is inhibited by quercetin in a HO1-dependent fashion (Nakamura et al., 2011). Furthermore, TGFβ-mediated collagen synthesis is impaired by HO1 in atrial fibroblast (Yeh et al., 2016). Heme impairs TGFβ production in human mononuclear cells in Zn/Cu superoxide dismutase-dependent manner (Andrade et al., 2010). These data show that heme can inhibit both TGFβ production and the signaling cascade downstream of TGFβ, thereby regulating TGFβ responsive genes such as collagen. In addition, TGFβ signaling was markedly inhibited by the ER stress inducer thapsigargin in NIH3T3 and oligodendroglial OLI-neu cell lines (Alevizopoulos et al., 1997; Ming et al., 2010). ER stressor thapsigargin inhibited collagen synthesis as well in chick embryo chondrocytes (Clark et al., 1994). The modulatory effect of heme on TGFβ synthesis and signaling including collagen production can be mediated either by the induction of HO1, SOD, or, similar to thapsigargin, in an ER stress catalyzed way. Active TGFβ and its receptors are limitedly expressed in isolated areas of advanced atherosclerotic lesions together with lower serum levels in atherosclerosis (Grainger et al., 1995; Bobik et al., 1999; McCaffrey et al., 1999). These findings suggest that heme induced ER stress might contribute to lower expression of TGFβ and collagen type I resulting in plaque instability. We may conclude that our new observation related to fibrogenesis and heme effects are strongly supported by other models.

We demonstrate for the first time that heme induces ER stress and UPR in HAoSMCs. Heme is such a robust stress for cells, that it is able to induce CHOP, a pro-apoptotic protein. At the same time we were able to show, that heme induced only a transient increase in ATF4 with a concomitant sustained expression of Grp78 and ATF6 activation indicating an adaptive response to ER stress. Parallel with that, sustained activation of HO1 and ferritin heavy chain were observed in HAoSMCs in response to heme.

## Conclusion

Our findings provide *in vivo* as well as *in vitro* evidence that heme triggers ER stress that might be a novel coordinated program of responses to heme: “heme induced endoplasmic reticulum” stress (HIER stress). We demonstrated, that the cytotoxic and harmful effects of heme were attenuated by Hpx and A1M through their heme binding capability. Our results support those efforts where Hpx and A1M could serve as therapeutic agents in various diseases where excess heme is present.

## Author Contributions

TG, DP, and AN designed and performed the experiments, analyzed the data. TG wrote the manuscript and contributed to study design. ZH performed immunohistochemistry. ZH and GM conducted pathological analyses of carotid arteries and advised histology. LP performed oxidized hemoglobin and heme measurements from human samples. PN provided human carotid artery specimens. BÅ and MG provided recombinant alpha-1-microglobulin and edited the manuscript. AS provided rabbit hemopexin, contributed to interpretation of the data and revised the manuscript. GB and JB contributed to the study design and edited the manuscript. GT and DP share first authorship.

## Conflict of Interest Statement

The authors declare that the research was conducted in the absence of any commercial or financial relationships that could be construed as a potential conflict of interest.
