# Supplementary material for: Heme Induces Endoplasmic Reticulum Stress (HIER Stress) in Human Aortic Smooth Muscle Cells
Source: Front Physiol. 2018 Nov 20;9:1595. doi: 10.3389/fphys.2018.01595 (PMC6255930; doi:10.3389/fphys.2018.01595)

Supplementary Material

**Heme induces endoplasmic reticulum stress (HIER stress) in human aortic smooth muscle cells.**

**Tamás Gáll, Dávid Pethő, Annamária Nagy, Zoltán Hendrik, Gábor Méhes, László Potor, Magnus Gram, Bo Åkerström, Ann Smith, György Balla and József Balla***

*** Correspondence: József Balla, balla@belklinika.com**

**Supplementary figure 1**


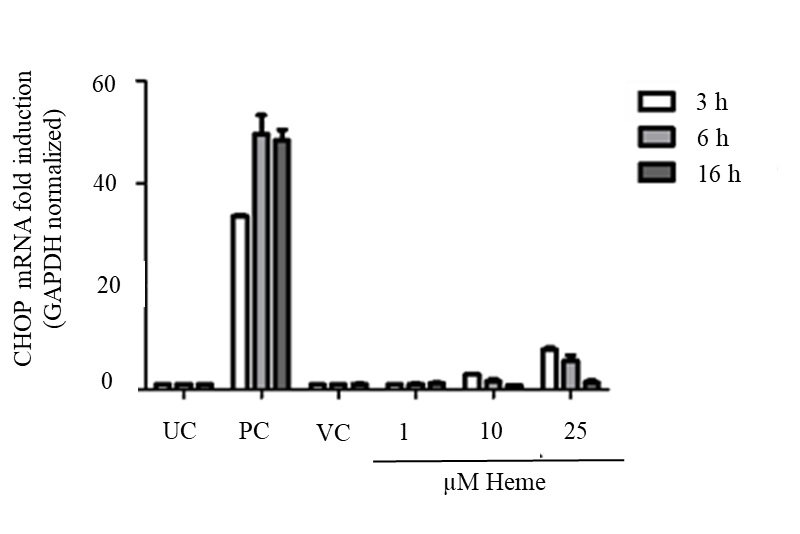


**Supplementary figure 1.** HAoSMCs were treated with various doses of heme (1,10, and 25 µM) or vehicle (20 mM NaOH) corresponding to highest heme dose (25 µM) in serum-free DMEM for 60 minutes, then medium was changed to DMEM with 10% FCS and antibiotics. ER stress markers were measured after 3, 6, or 16 hours. Thapsigargin (1 µM) treated cells were used as positive control**)**. Relative expressions of CHOP mRNA levels were determined by qRT-PCR, normalized to GAPDH.

Supplememtary figure 2


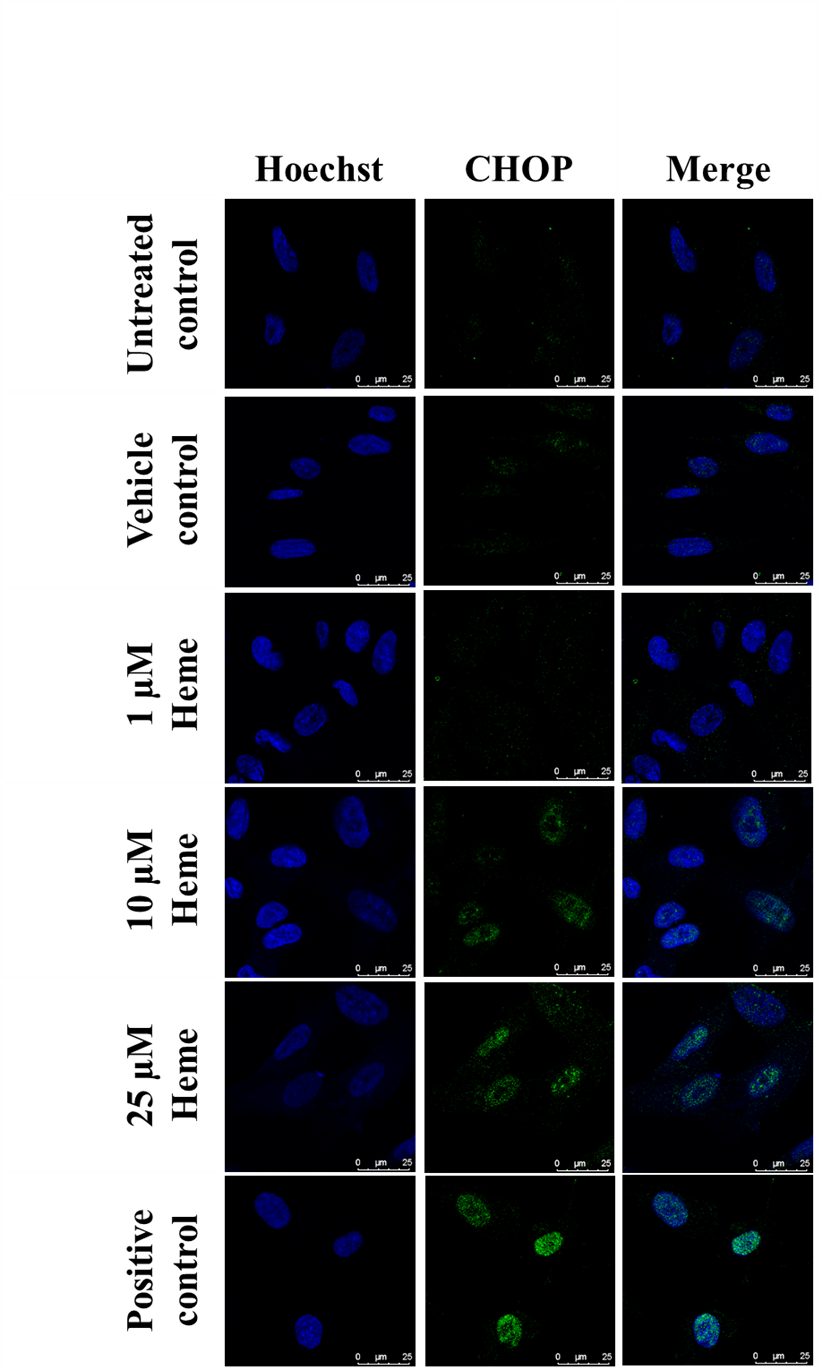


Supplementary figure 2. Analysis of CHOP expression and nuclear translocation after heme chellenge. Cells were treated with vehicle or various doses of heme (1,10, and 25 µM) for 60 min in serum-free DMEM then the medium was changed to DMEM+10% FCS+antibiotics. CHOP expression and localization was followed by confocal immunofluorescent microscopy three hours after the medium replacement. Thapsigargin (1 µM) treated cells were used as positive control.

Supplementary figure 3


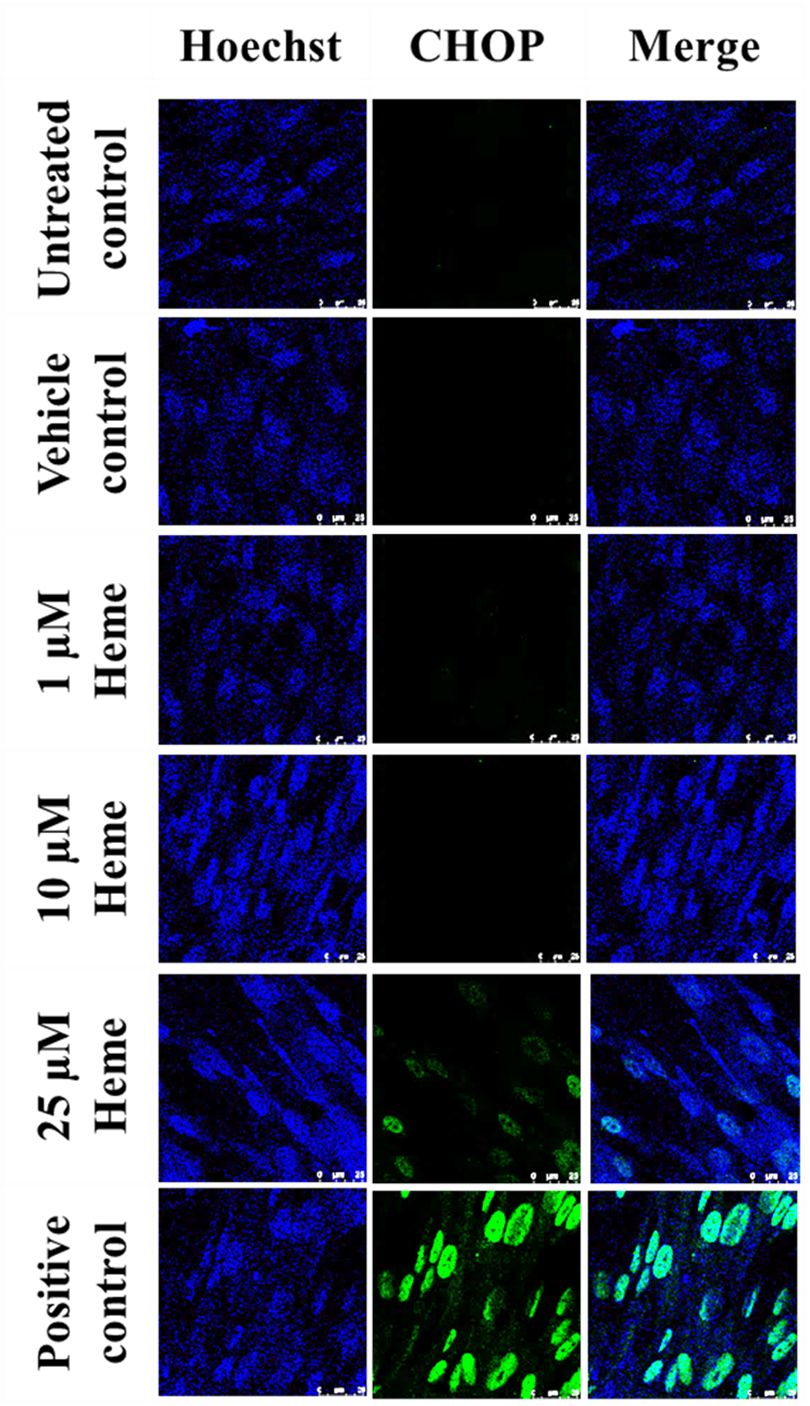


Supplementary figure 3. Analysis of CHOP expression and nuclear translocation after heme chellenge. Cells were treated with vehicle or various doses of heme (1,10, and 25 µM) for 60 min in serum-free DMEM then the medium was changed to DMEM+10% FCS+antibiotics. CHOP expression and localization was followed by confocal immunofluorescent microscopy six hours after the medium replacement. Thapsigargin (1 µM) treated cells were used as positive control.

Supplementary figure 4


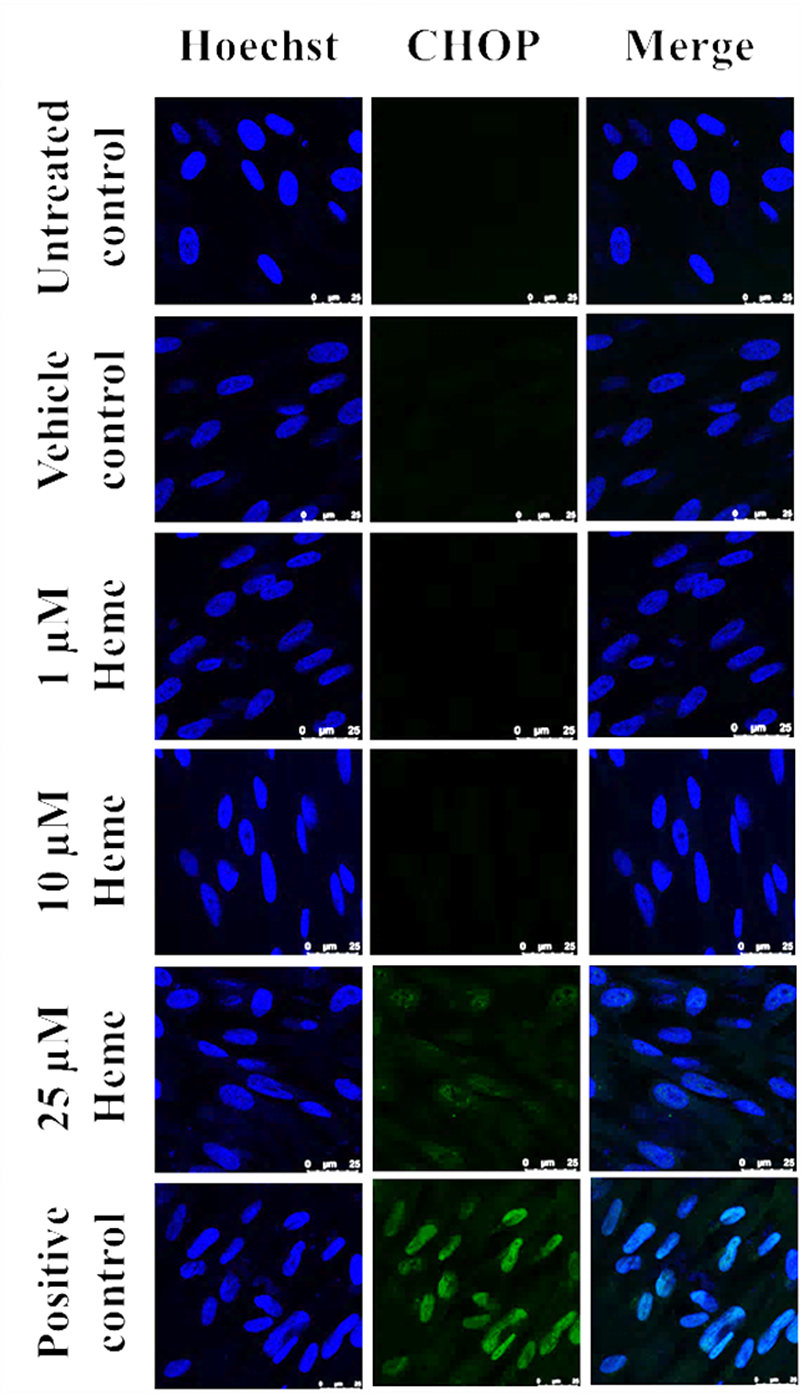


Supplementary figure 4. Analysis of CHOP expression and nuclear translocation after heme chellenge. Cells were treated with vehicle or various doses of heme (1,10, and 25 µM) for 60 min in serum-free DMEM then the medium was changed to DMEM+10% FCS+antibiotics. CHOP expression and localization was followed by confocal immunofluorescent microscopy sixteen hours after the medium replacement. Thapsigargin (1 µM) treated cells were used as positive control.

**Supplementary figure 5**
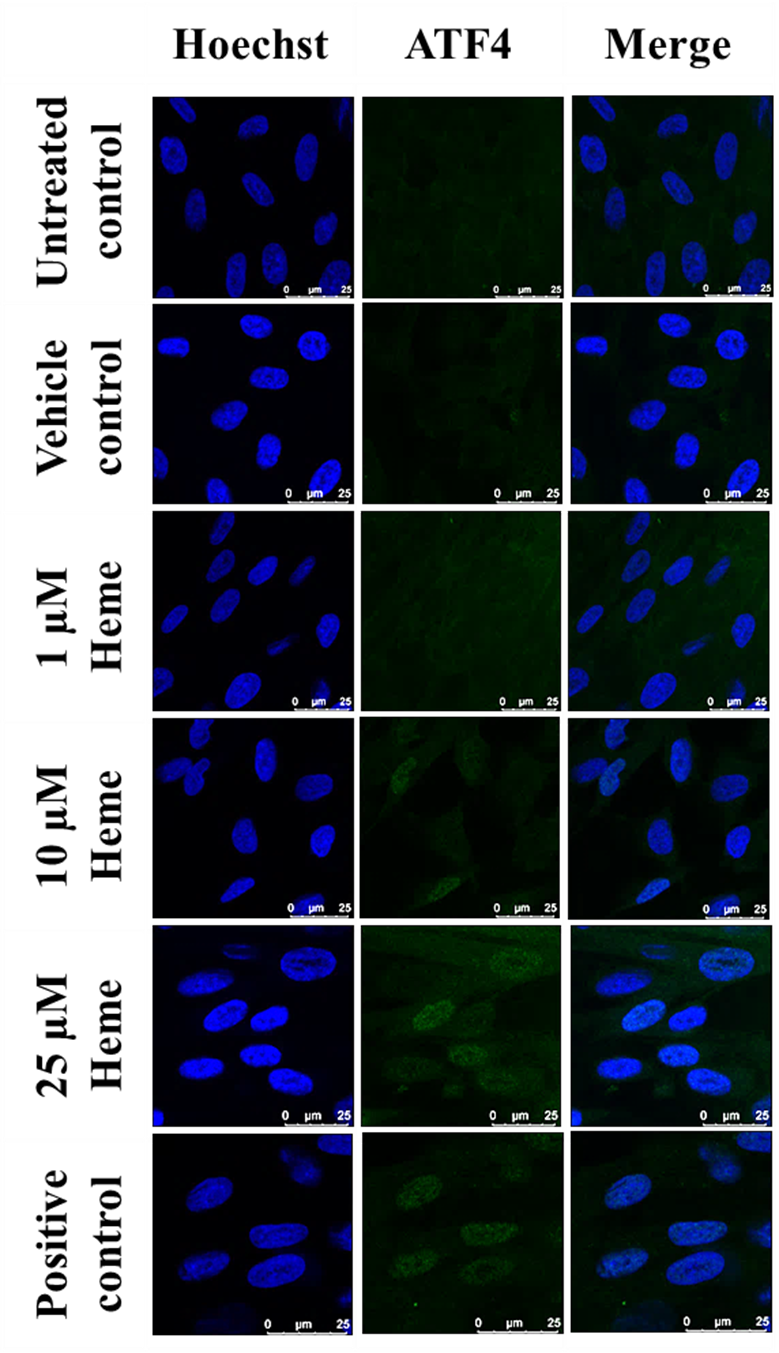


**Supplementary figure 5.** Analysis of ATF4 expression and nuclear translocation after heme chellenge. Cells were treated with vehicle or various doses of heme (1,10, and 25 µM) for 60 min in serum-free DMEM then the medium was changed to DMEM+10% FCS+antibiotics. ATF4 expression and localization was followed by confocal immunofluorescent microscopy three hours after the medium replacement. Thapsigargin (1 µM) treated cells were used as positive control.

Supplementary figure 6


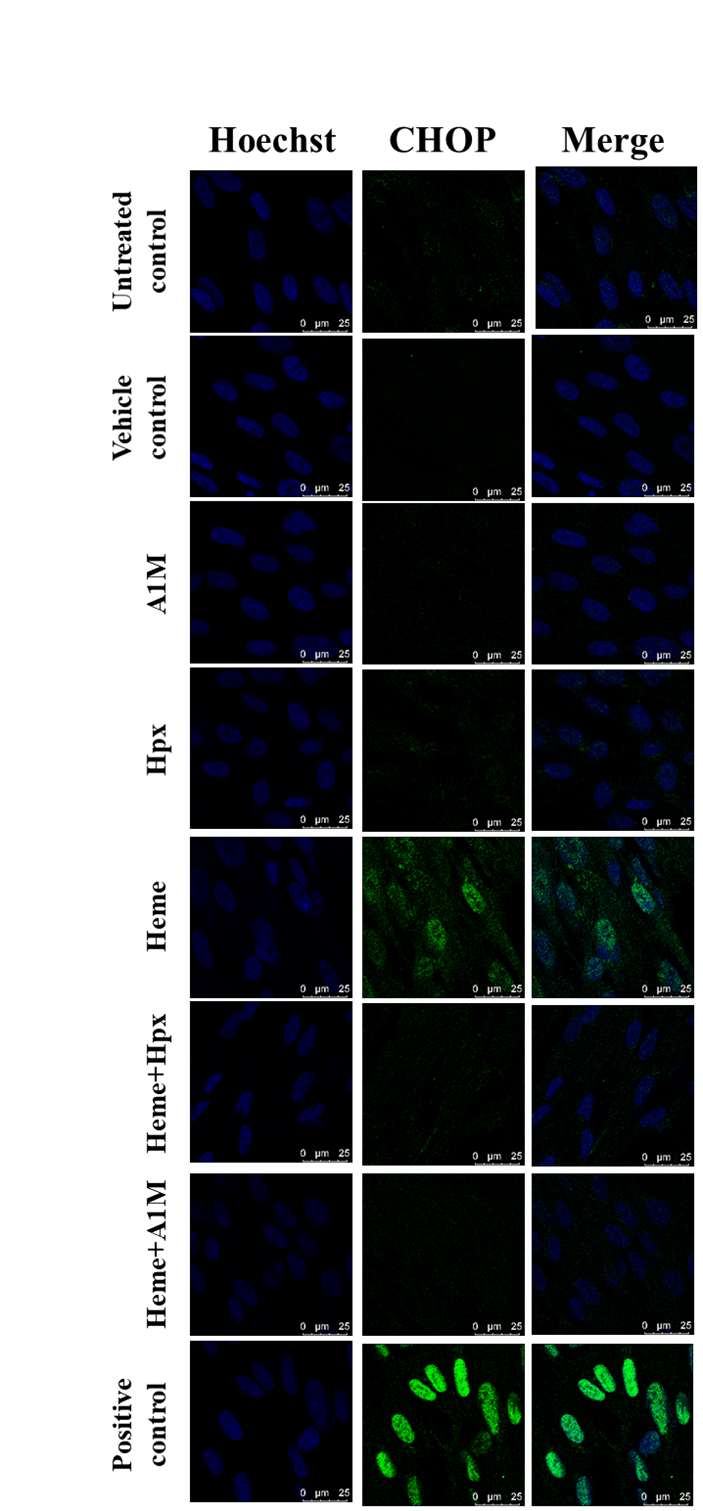


Supplementary figure 6. Analysis of CHOP expression and nuclear translocation after heme chellenge alone or in a combination with the heme scavenger hemopexin (Hpx) or recombinant alpha-1-microglobulin (rA1M). Cells were treated with heme (25 µM) or heme (25 µM)+Hpx (25µM) or heme (25 µM)+rA1M (12.5 µM) for 60 min in serum-free DMEM then the medium was changed to DMEM+10% FCS+antibiotics. CHOP expression and localization was followed by confocal immunofluorescent microscopy three hours after the medium replacement. Thapsigargin (1 µM) treated cells were used as positive control.

Supplementary figure 7


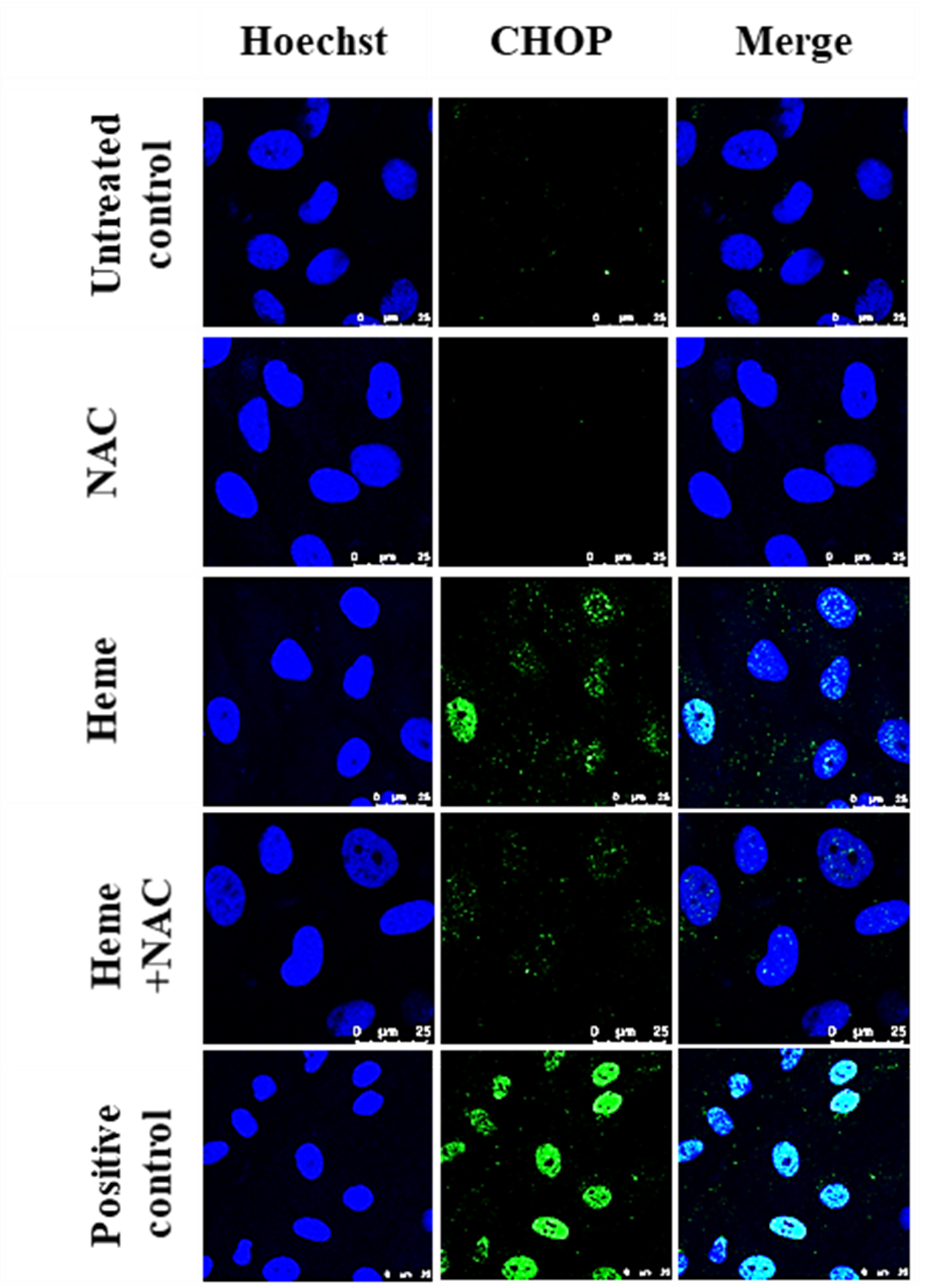


Supplementary figure 7. Analysis of CHOP expression and nuclear translocation after heme chellenge alone or in a combination with the antioxidant N-acetyl cysteine (NAC; 10 mM). CHOP expression and localization was followed by confocal immunofluorescent microscopy three hours after the medium replacement. Thapsigargin (1 µM) treated cells were used as positive control.

**Supplementary figure 8.**

**
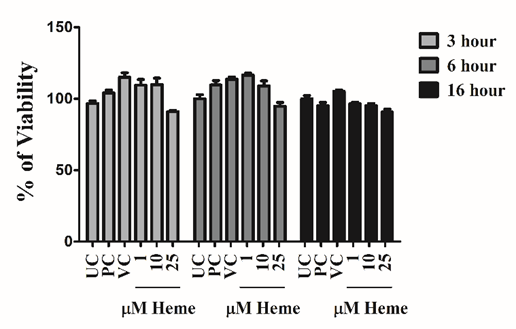
**

**Supplementary figure 8.** Cells were treated with various doses of heme (1, 10, and 25 µM) or thapsigargin (1 µM) as mentioned above. Cell viability was analyzed by MTT ((3-[4,5-dimethylthiazol-2-yl]-2,5 diphenyl tetrazolium bromide) assay. Results are presented as mean ± SD of five independent experiments.

Original blots

**ATF6 immunoblot heme dose (3,6,16h and Alpha-1-microglobulin and Hemopexin inhibition 3h)**


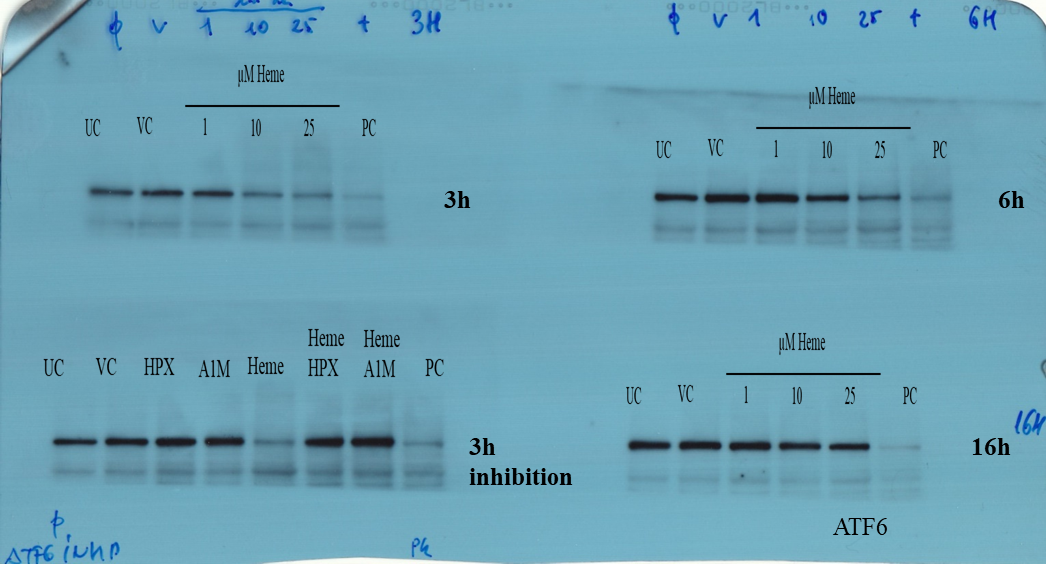


**Grp78 immunoblot heme dose 3h**


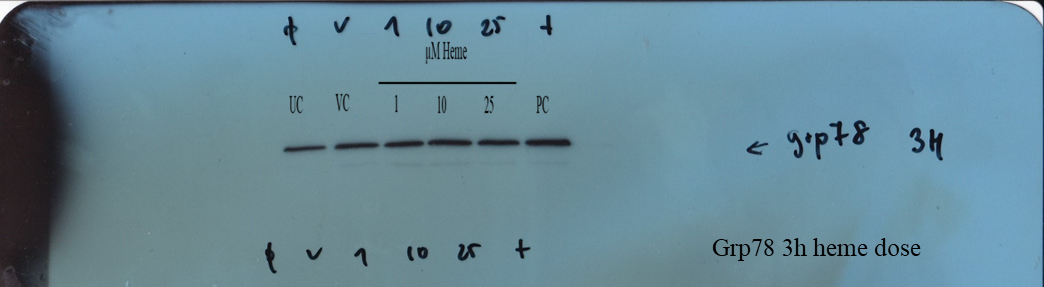


**Phosphor-eIF2α and ATF4 immunoblot, heme dose, 3h**


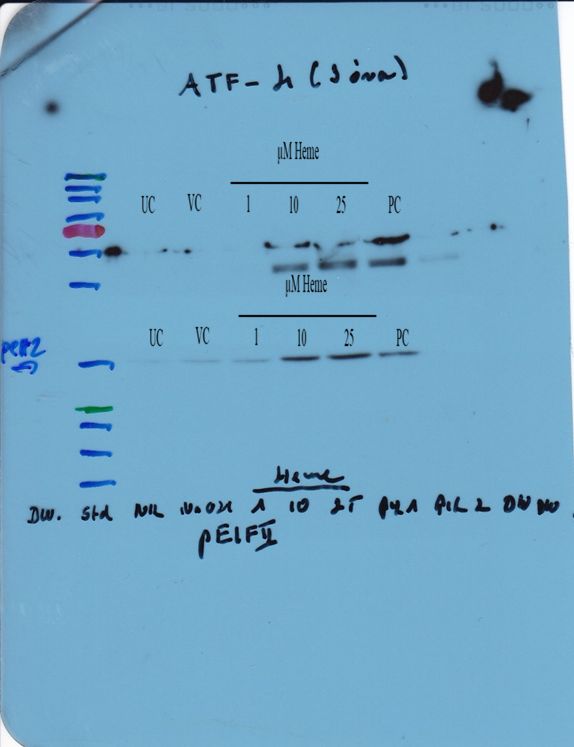


**HO1 and FTH, heme dose, 3h**


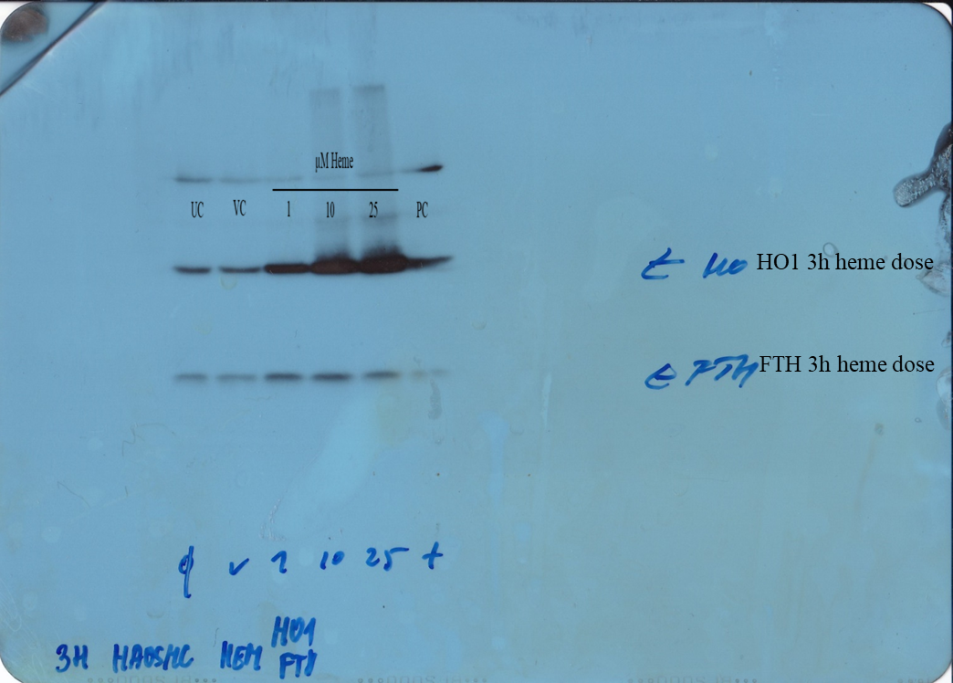


**eIF2α and GAPDH, heme dose, 3h**


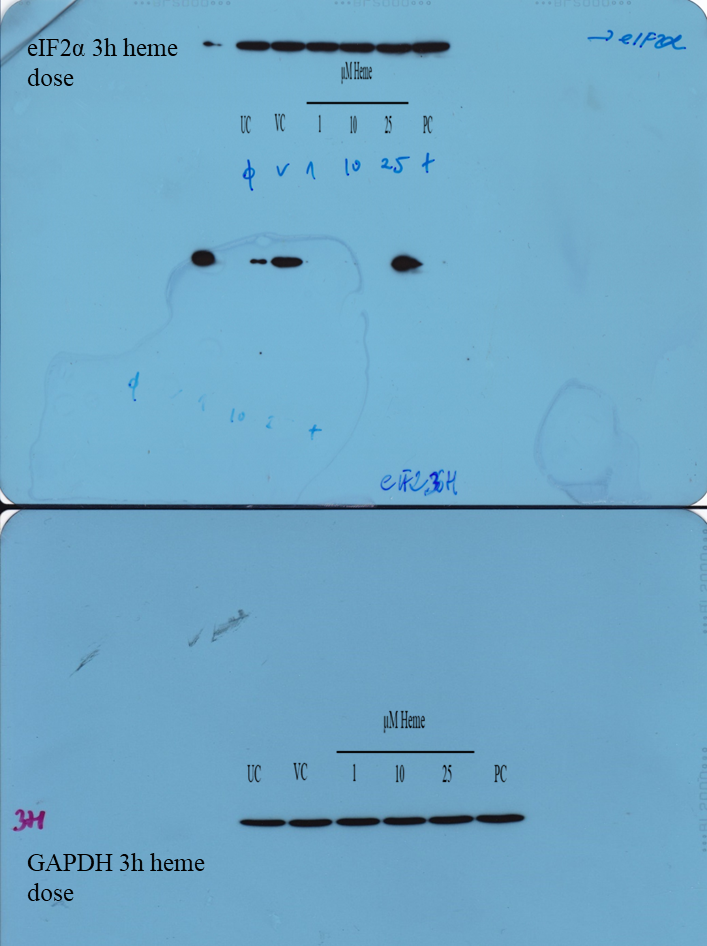


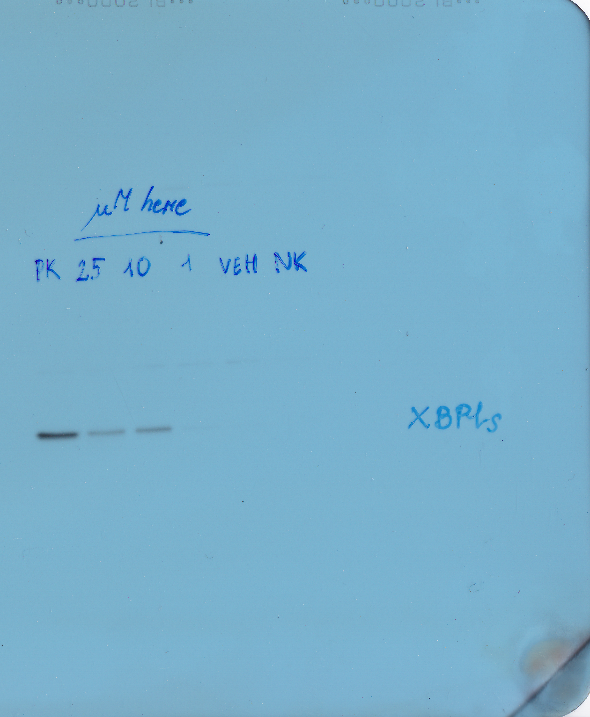


XBP1 3H heme dose


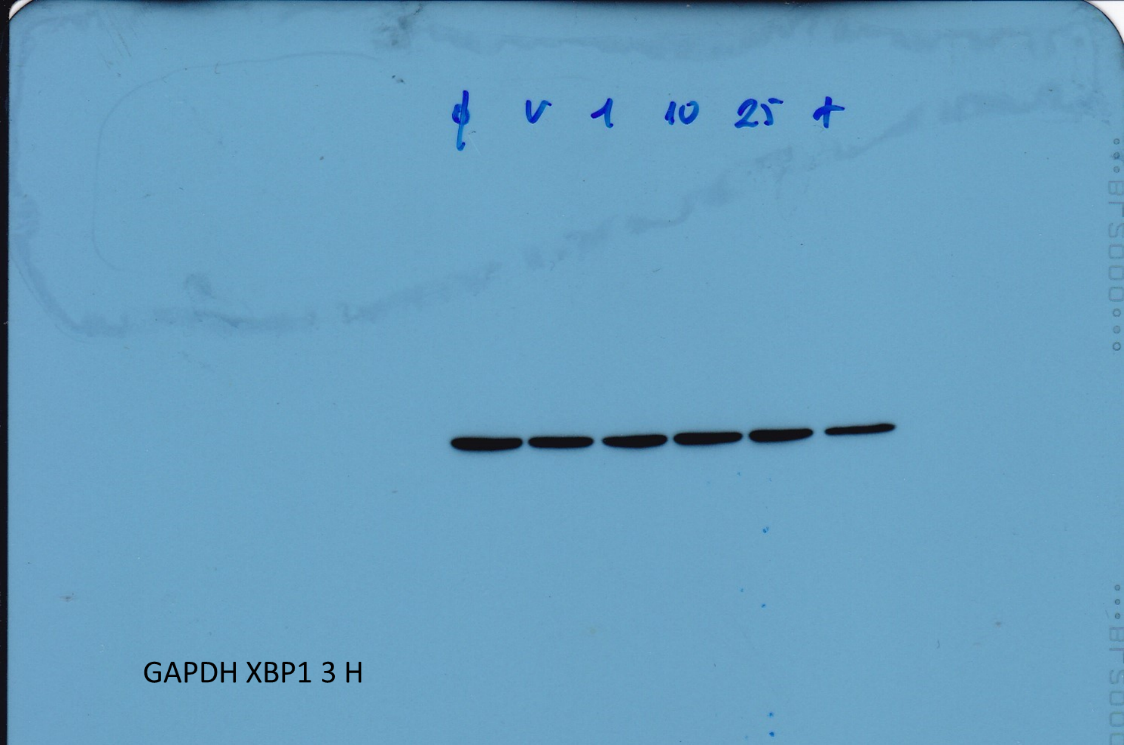


**ATF6 immunoblot heme dose (3,6,16h and Alpha-1-microglobulin and Hemopexin inhibition 3h)**


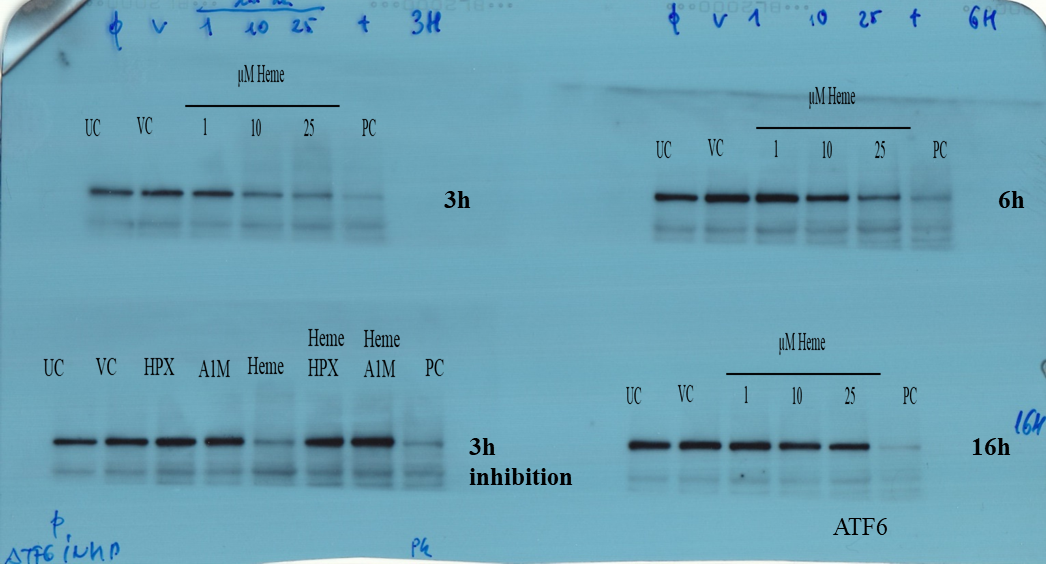


**ATF4 immunoblot 3h A1M and Hpx inhibition**


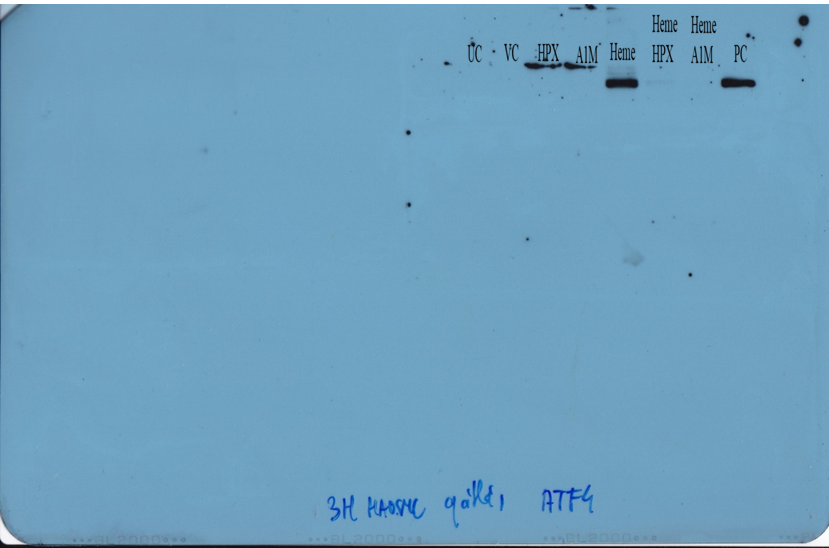


**Phospho eiF2α immunoblot 3h A1M and Hpx inhibition**


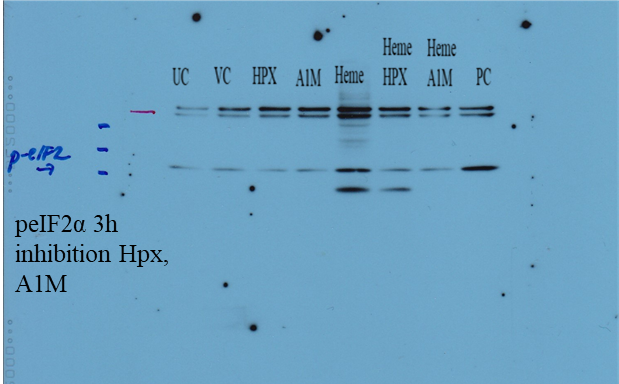


**eiF2α immunoblot 3h A1M and Hpx inhibition**


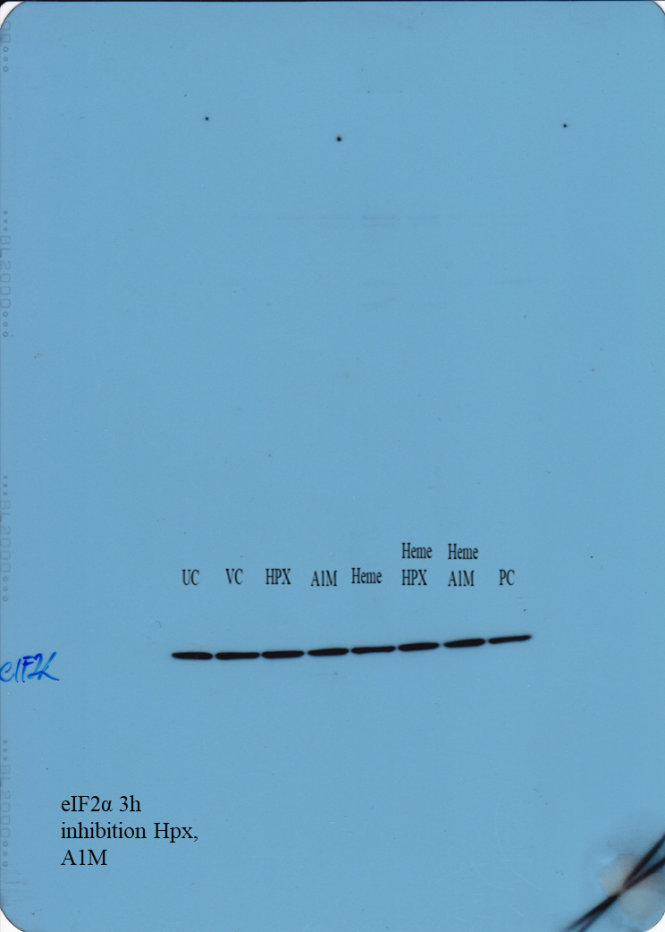


**HO1 immunoblot 3h A1M and Hpx inhibition**


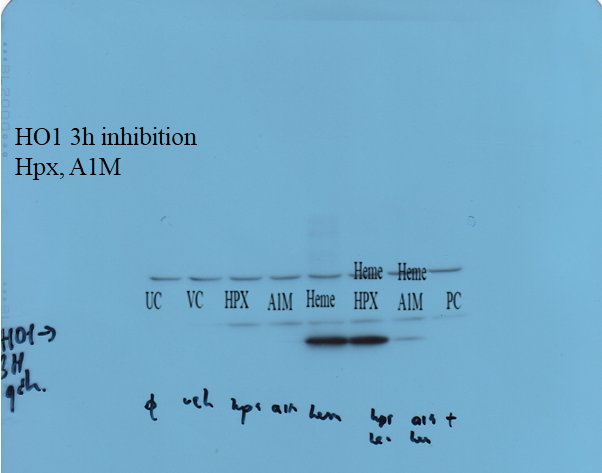


**FTH immunoblot 3h A1M and Hpx inhibition**


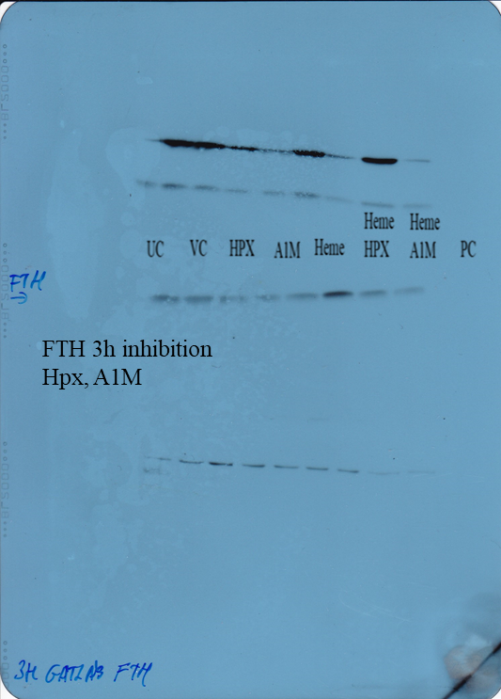


**XBP1s 3h inhibition**


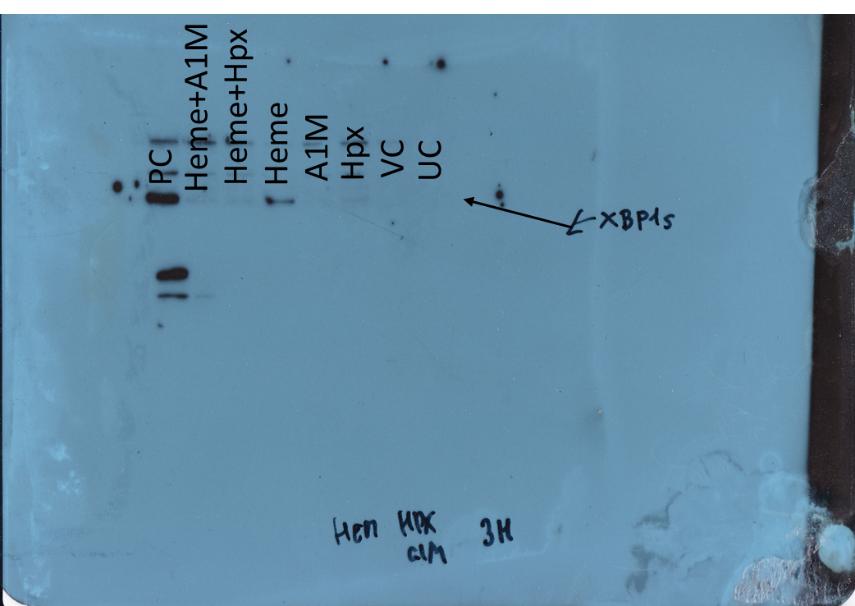


**GAPDH immunoblot 3h A1M and Hpx inhibition**

**
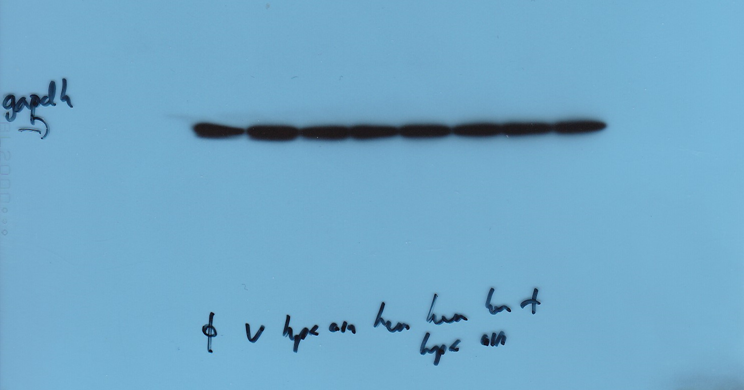
**

**ATF6 immunoblot, heme dose, 6h**


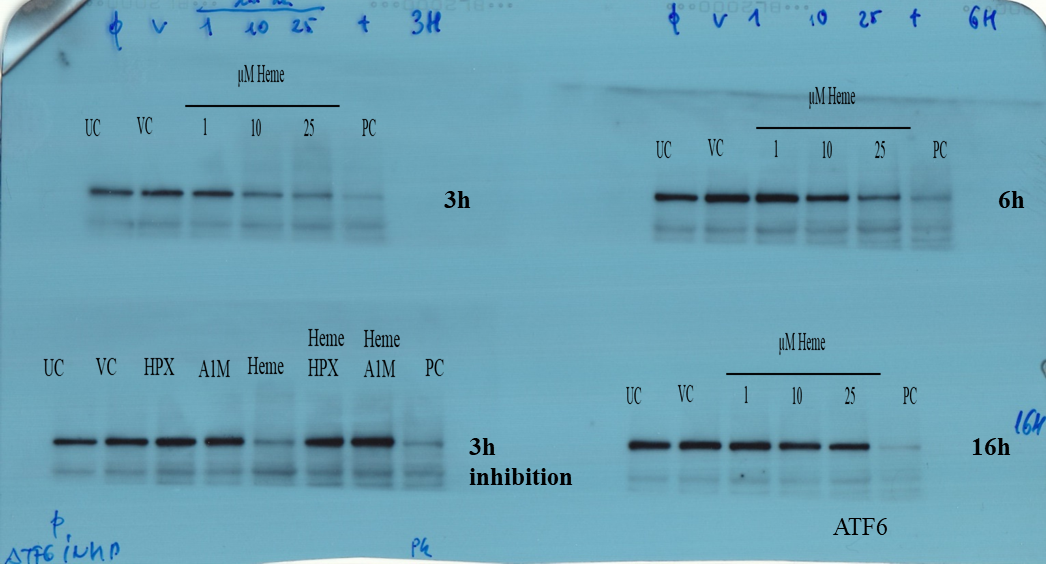


**Grp78 immunoblot, heme dose, 6h**

**
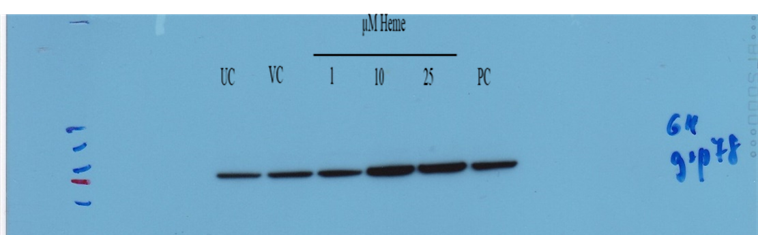
**

**XBP1s immunoblot, heme dose, 6h**

**
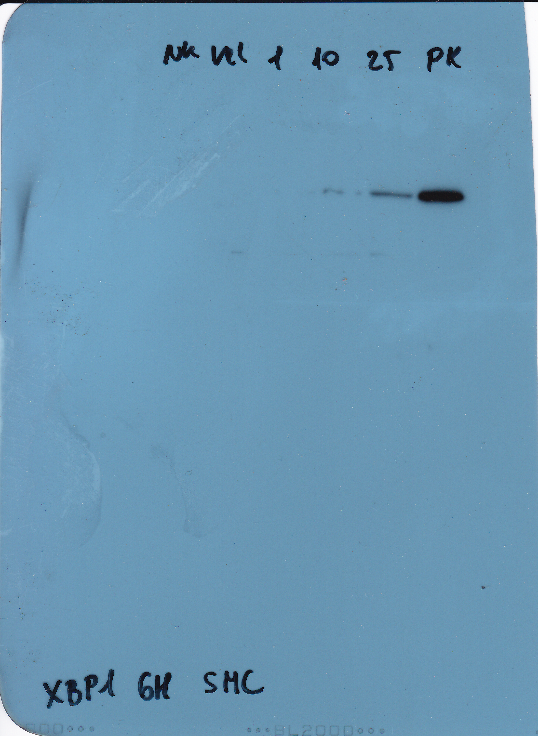
**

**GAPDH immunoblot, heme dose, 6h**

**
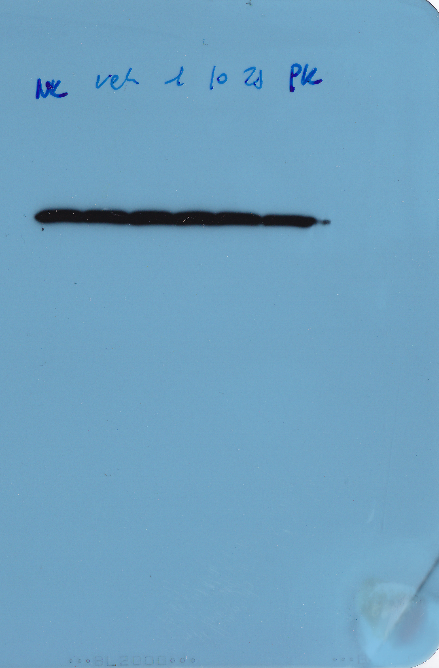
**

**ATF4 immunoblot, heme dose, 6h**


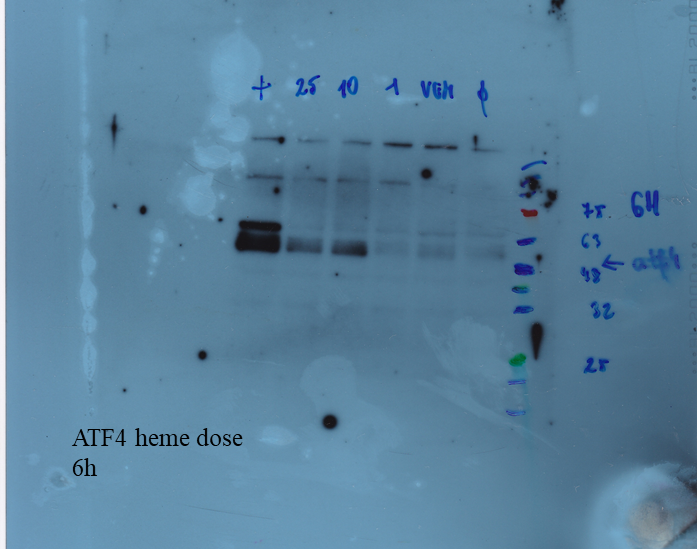


**Phospho-eiF2α immunoblot, heme dose, 6h**


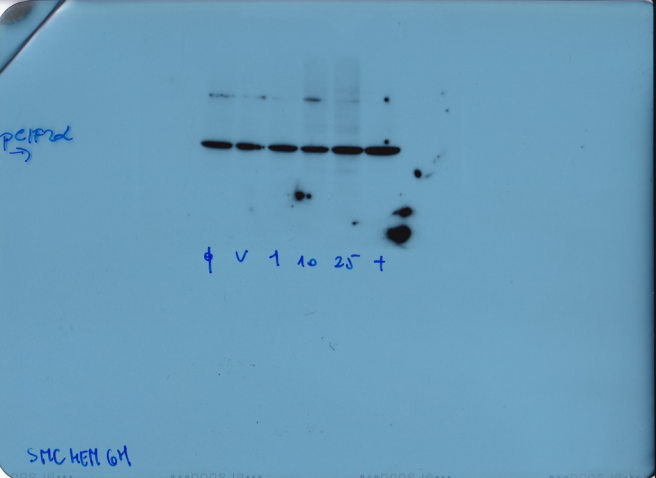


**eiF2α immunoblot, heme dose, 6h**


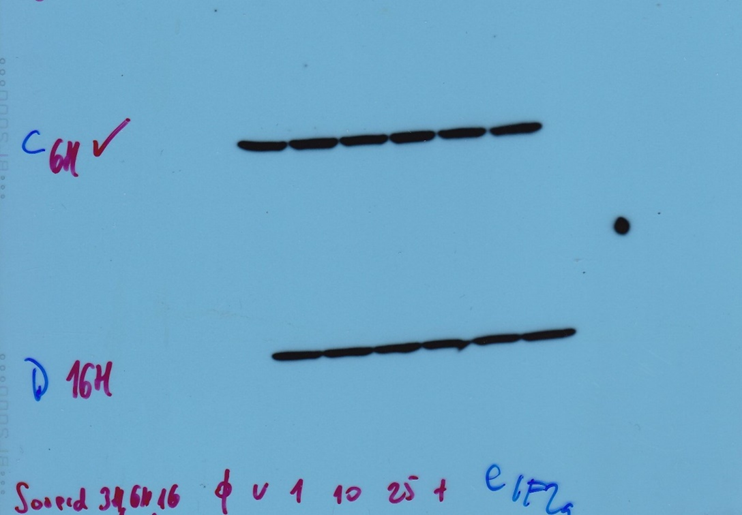


**HO1 immunoblot, heme dose, 6h**


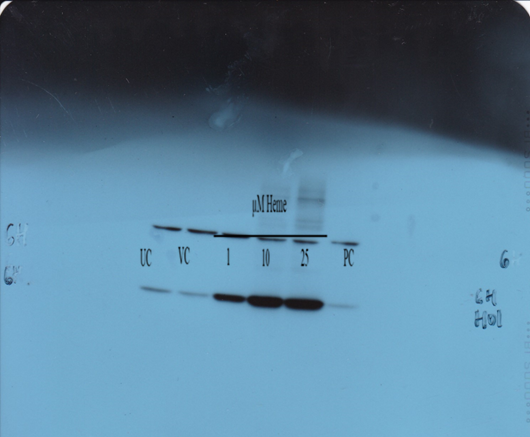


**FTH heme dose, 6h**


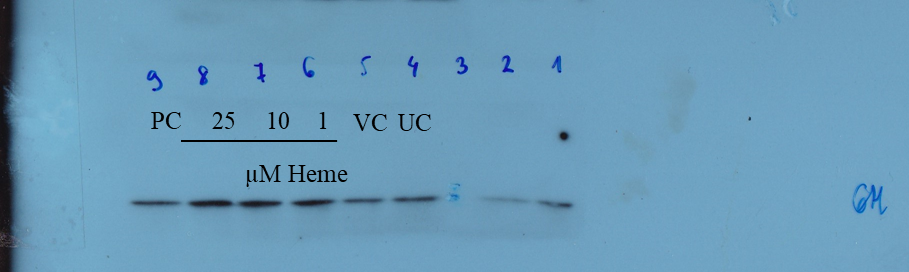


**GAPDH heme dose, 6h**

**
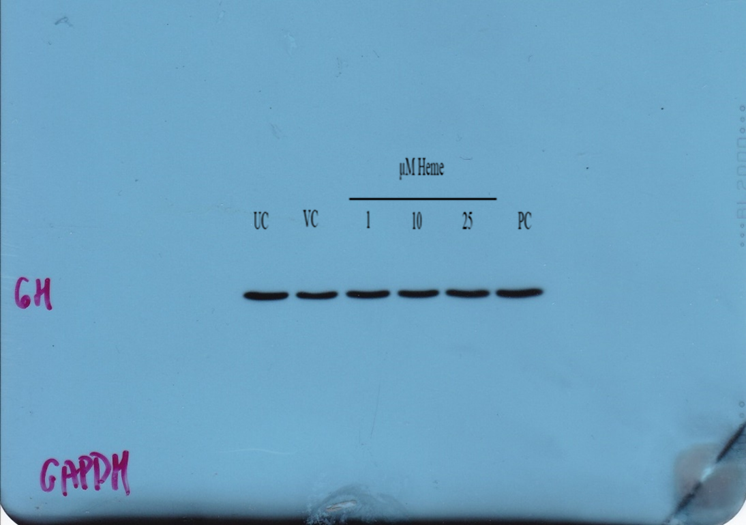
**

**ATF6 immunoblot, heme dose, 16h**


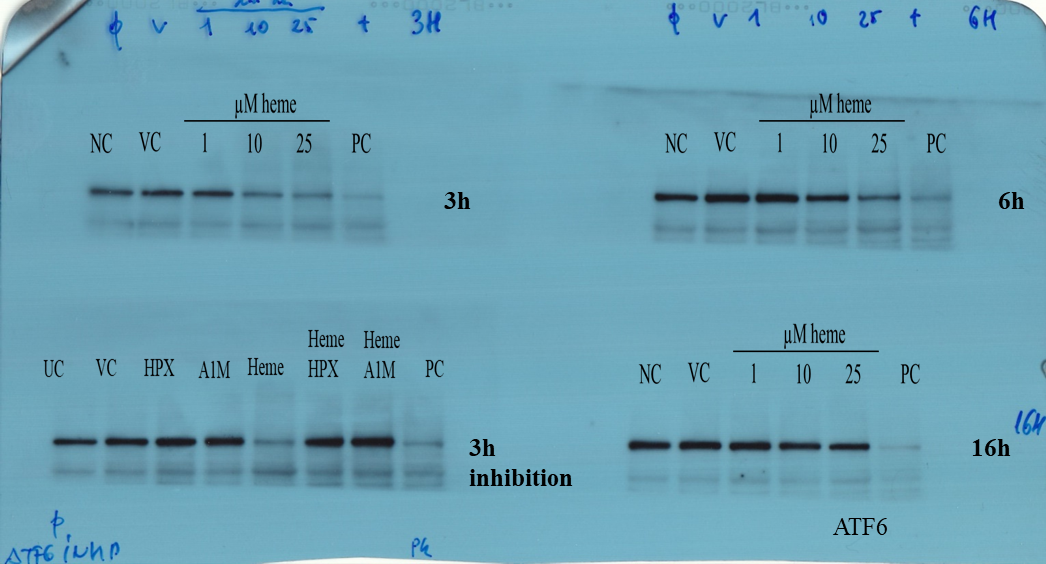


**Grp78 immunoblot, heme dose, 16h**


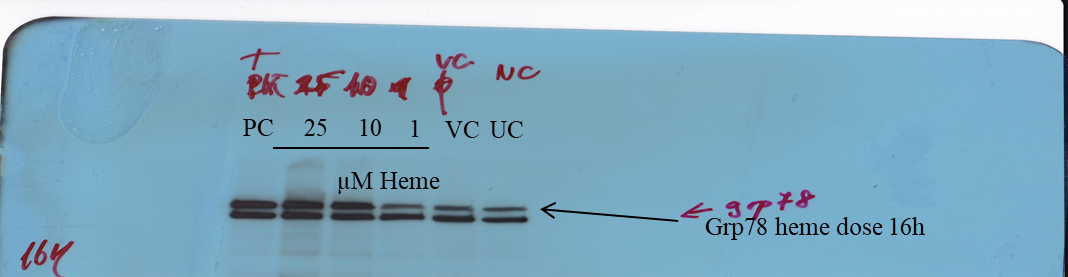


**ATF4 immunoblot, heme dose, 16h**


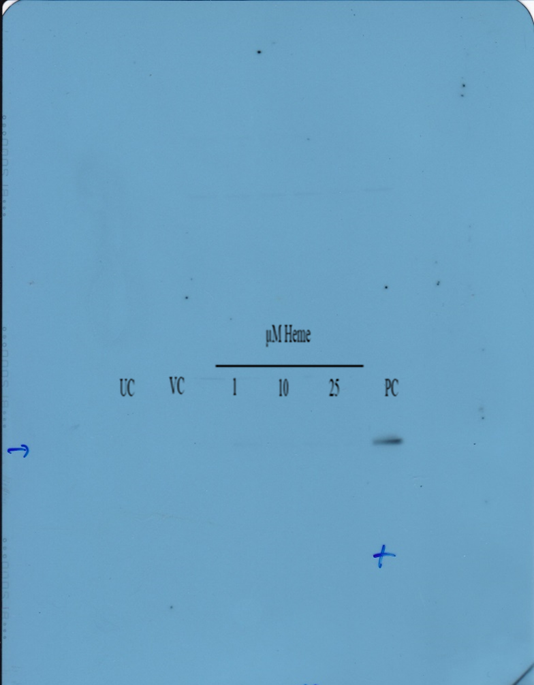


**XBP1s immunoblot 16h**

**
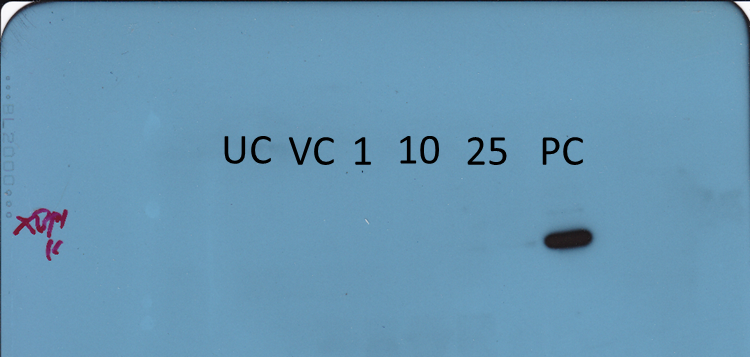
**

**GAPDH for XBP1s**

**
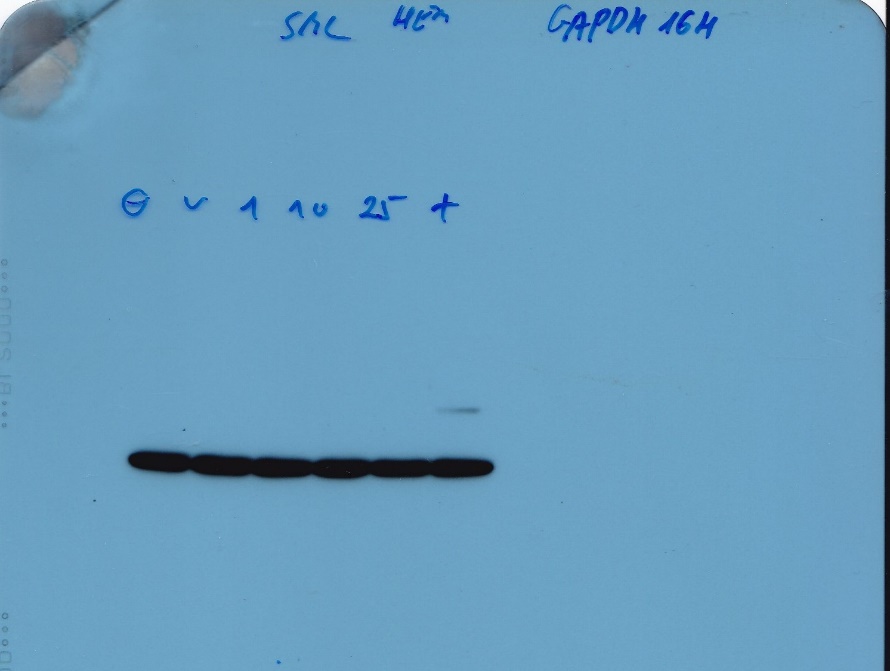
**

**Phospho-eiF2α immunoblot, heme dose, 16h**


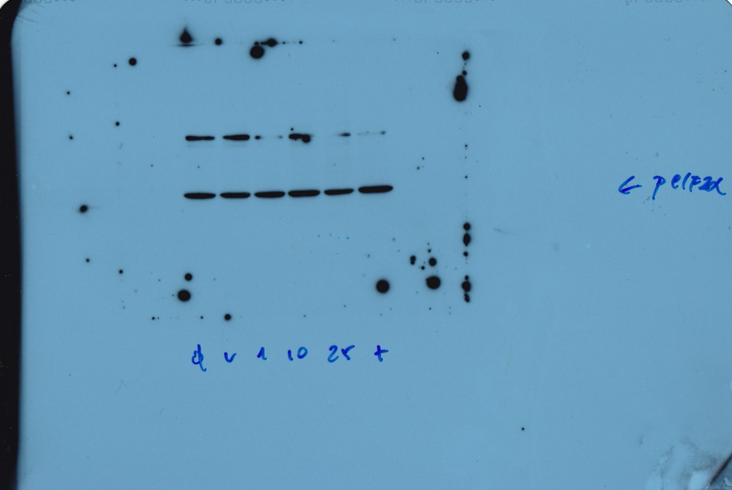


**eiF2α immunoblot, heme dose, 16h**


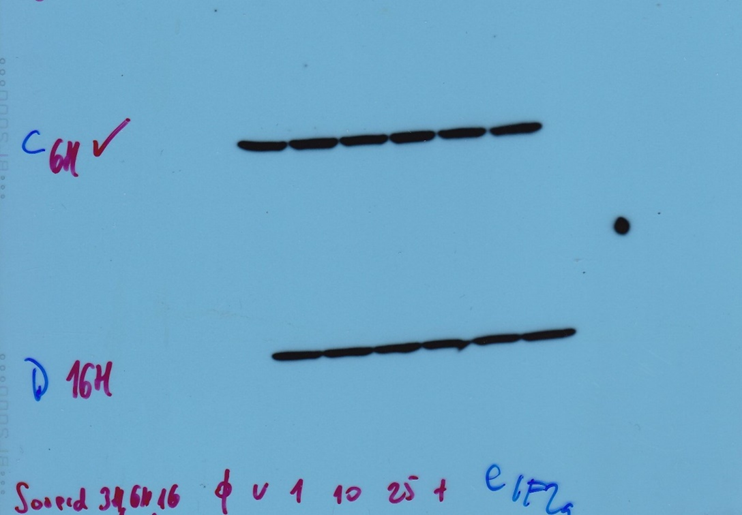


**HO1 immunoblot, heme dose, 16h**


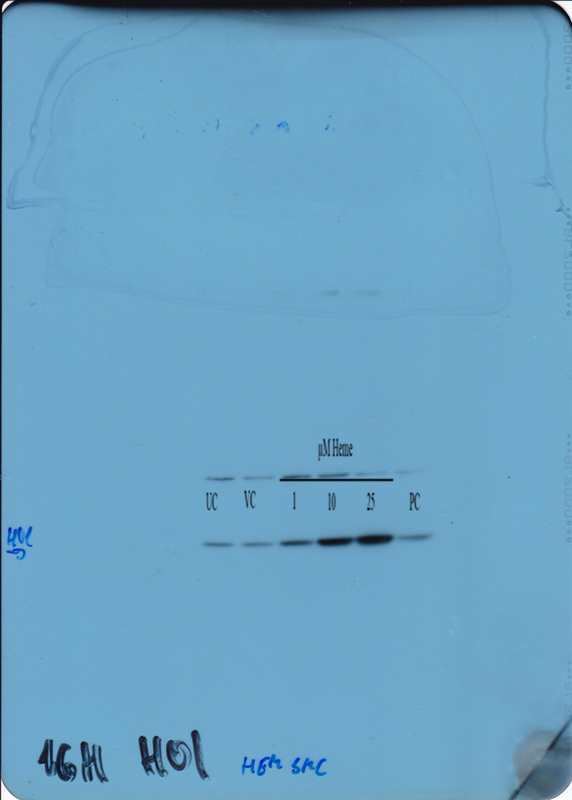


**FTH immunoblot, heme dose, 16h**

**
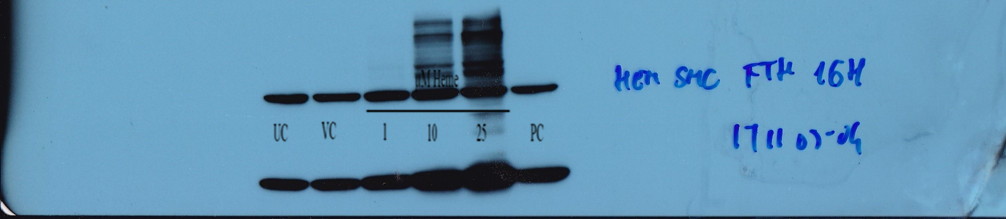
**

**GAPDH immunoblot, heme dose, 16h**


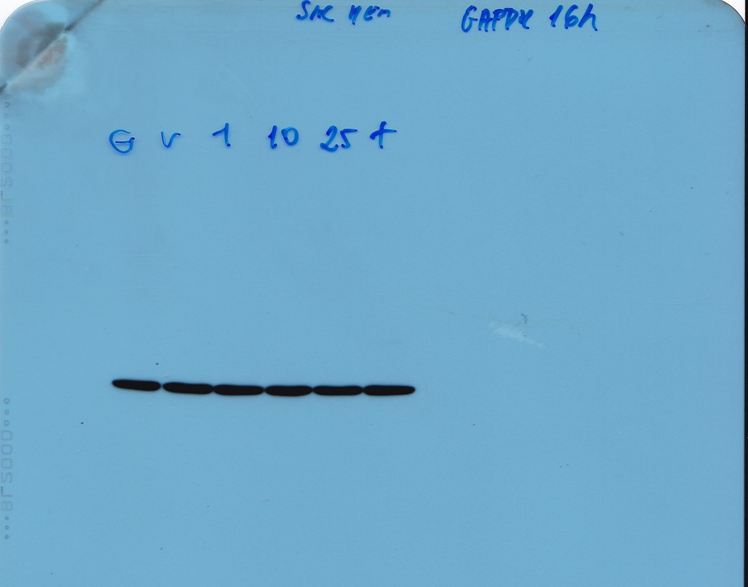


**Grp78 immunoblot, A1M and HPX inhibition, 16h**


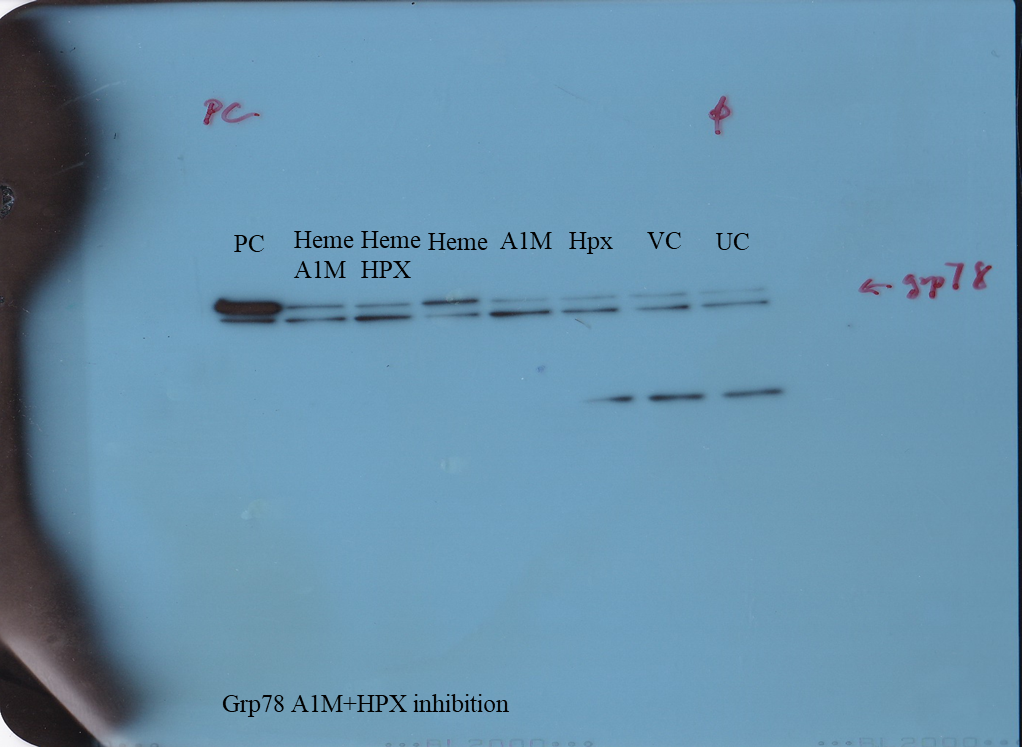


**HO1 immunoblot, A1M and HPX inhibition, 16h**


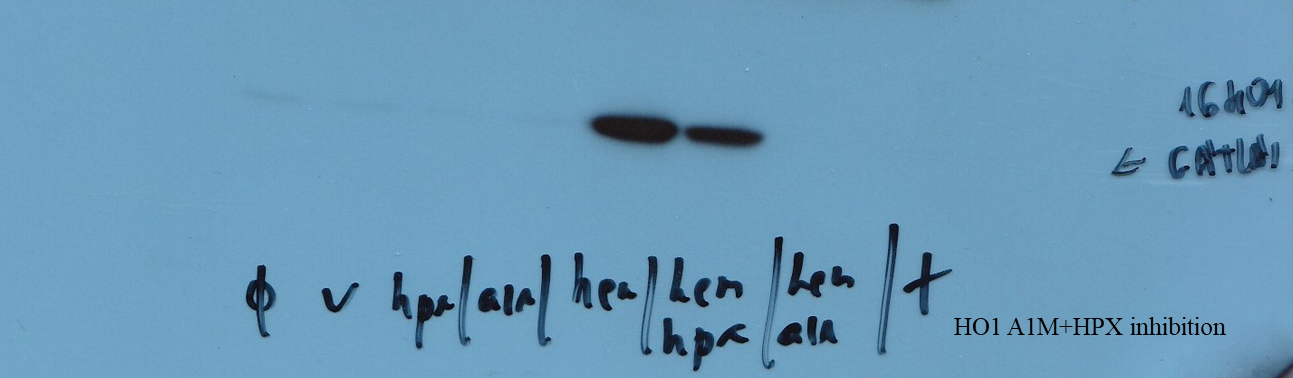


**FTH immunoblot, A1M and HPX inhibition, 16h**


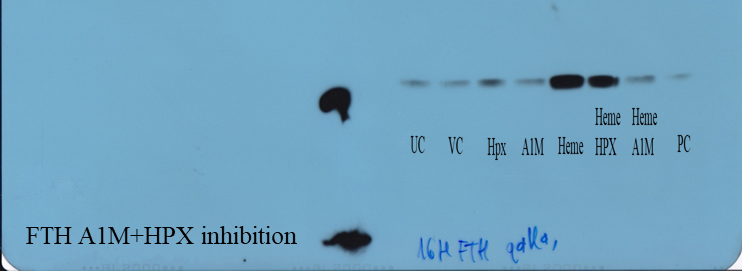


**GAPDH immunoblot, A1M and HPX inhibition, 16h**


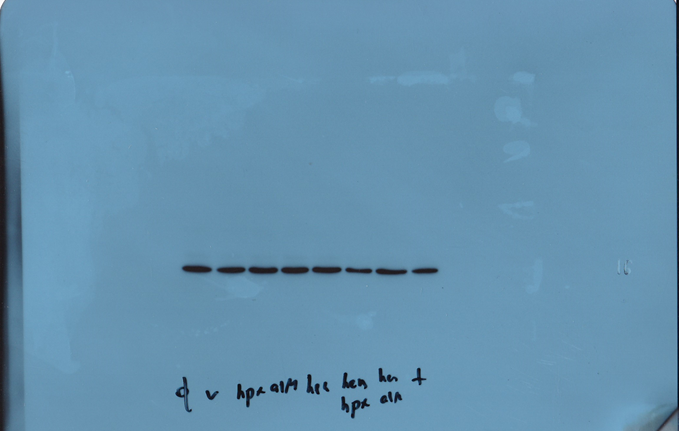


**XBP1u/XBP1s splicing analysis, agarose gel electrophoresis 3h, 6h**


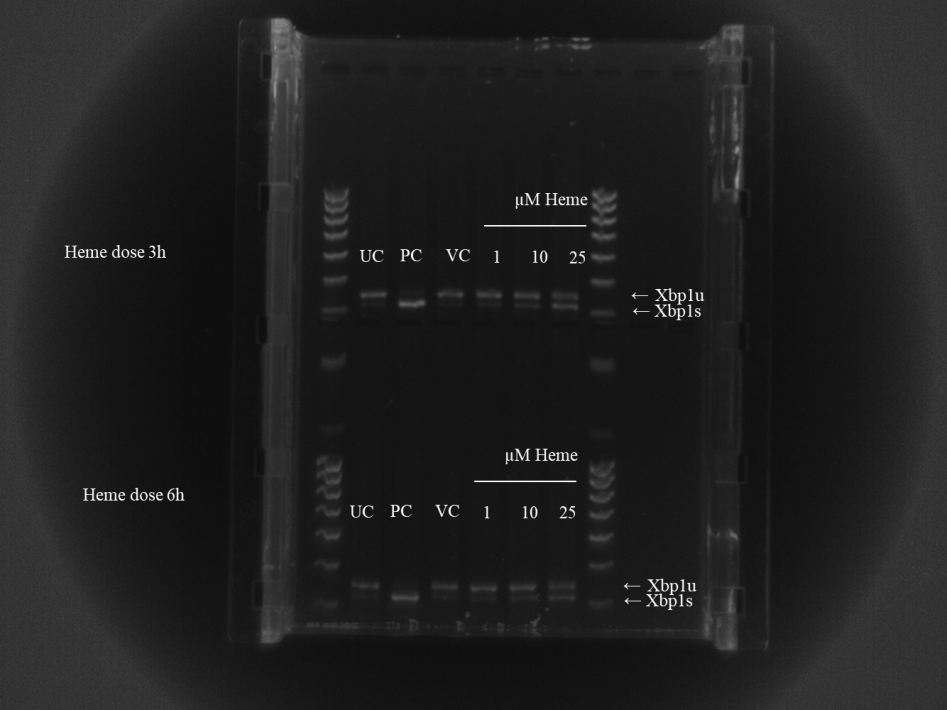


**XBP1u/XBP1s splicing analysis, agarose gel electrophoresis 16h**

**
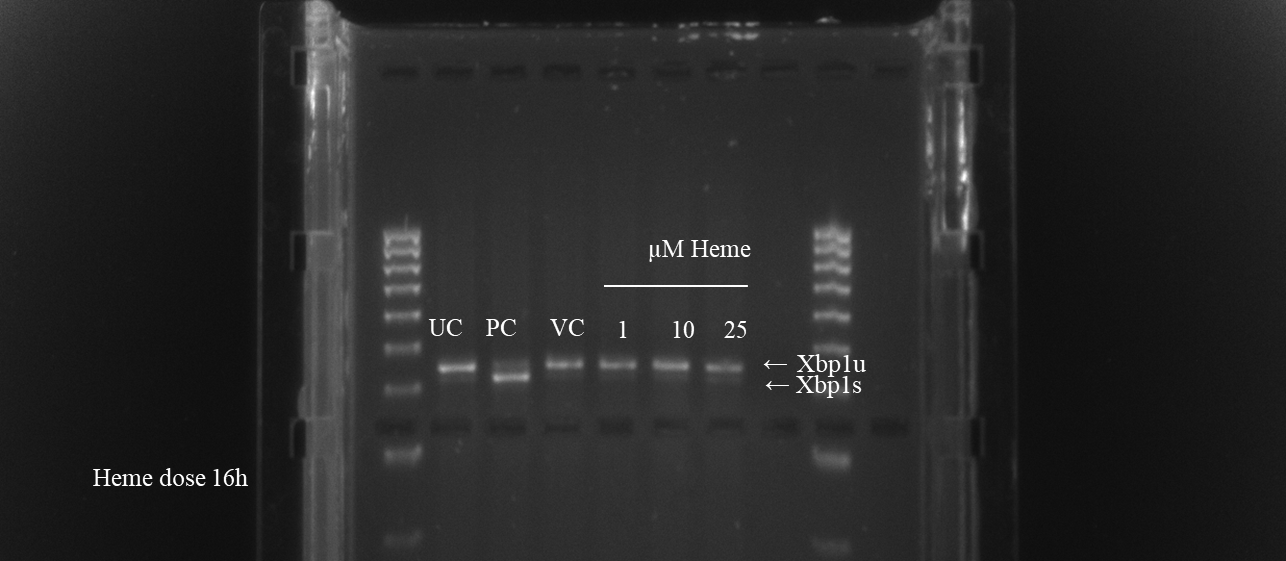
**

**GAPDH, agarose gel electrophoresis 3h, 6h**

**
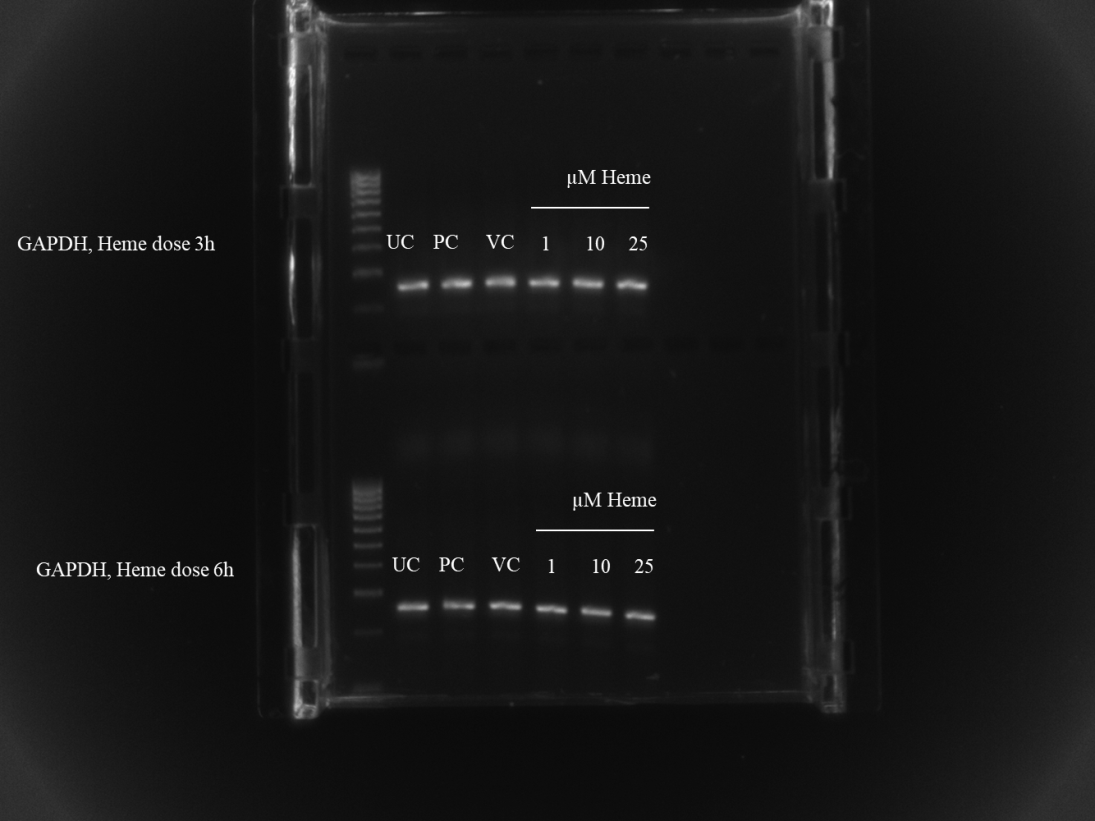
**

**GAPDH, agarose gel electrophoresis 16h**


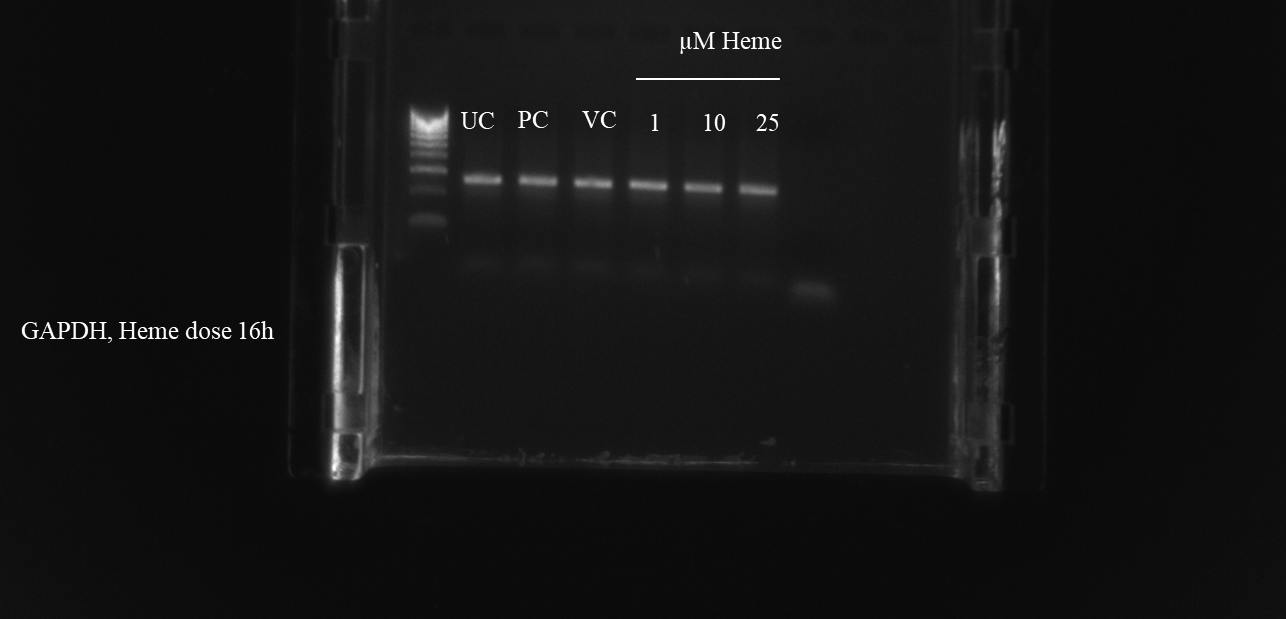


**XBP1u/XBP1s splicing analysis, agarose gel electrophoresis, A1M inhibition 3h**

**
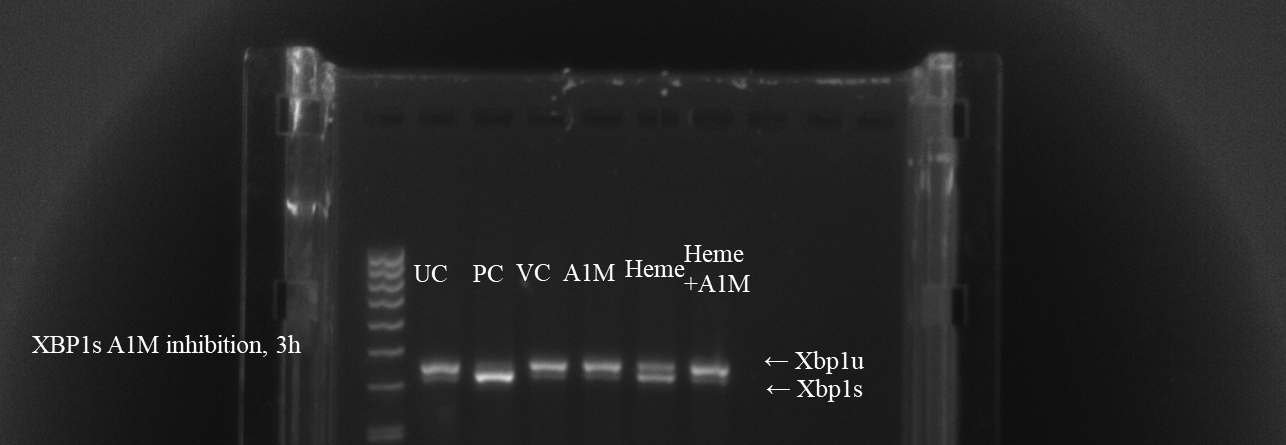
**

**GAPDH, agarose gel electrophoresis, A1M inhibition 3h**

**
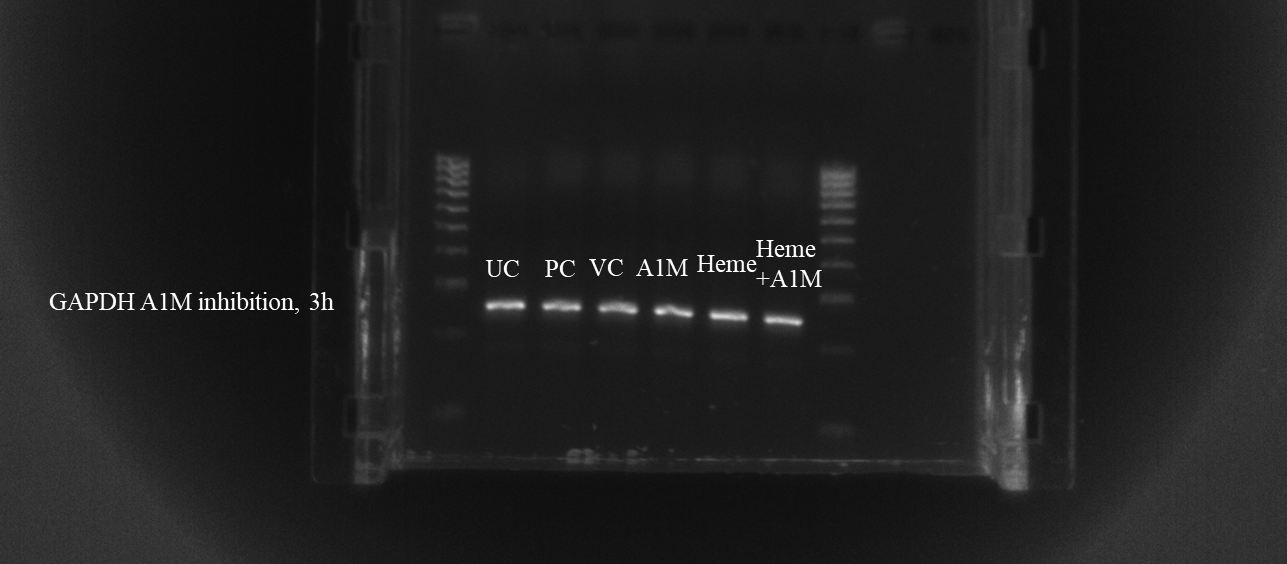
**

**XBP1u/XBP1s splicing analysis, agarose gel electrophoresis, Hpx inhibition 3h**


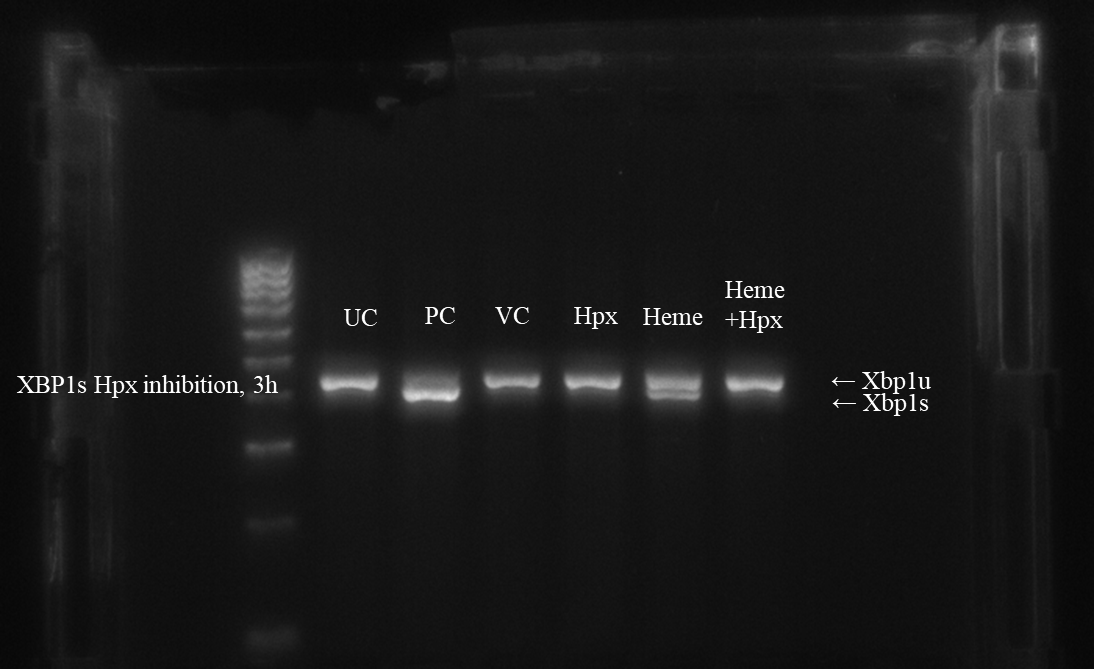


**GAPDH, agarose gel electrophoresis, Hpx inhibition 3h**


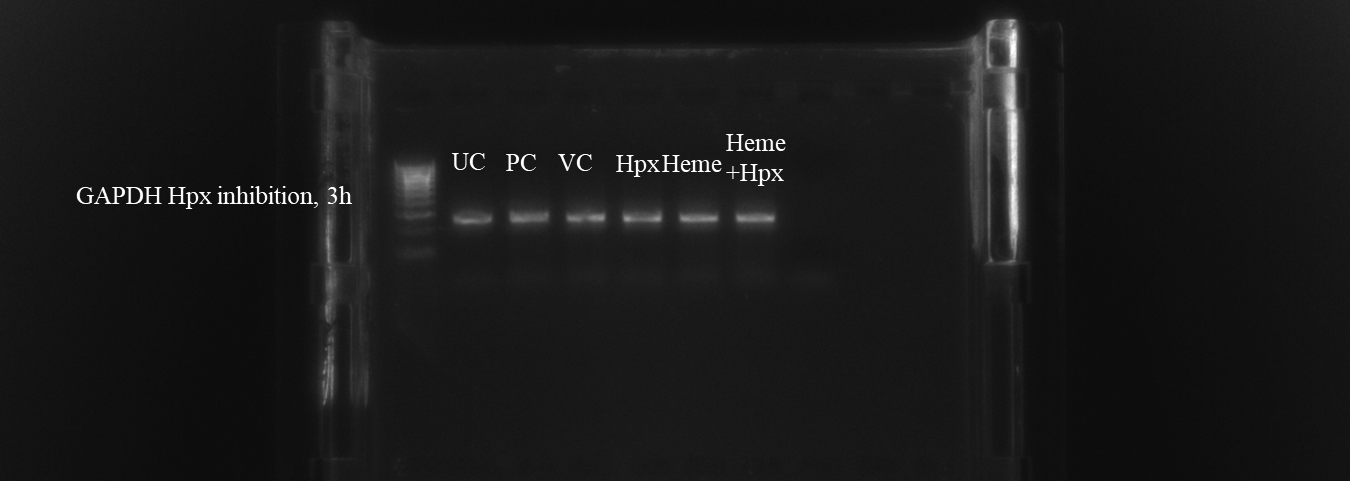

Supplement: Supplementary file 1 [file Table_1.docx]
